# Comorbidity between Alzheimer’s disease and major depression: a behavioural and transcriptomic characterization study in mice

**DOI:** 10.1186/s13195-021-00810-x

**Published:** 2021-04-02

**Authors:** Ana Martín-Sánchez, Janet Piñero, Lara Nonell, Magdalena Arnal, Elena M. Ribe, Alejo Nevado-Holgado, Simon Lovestone, Ferran Sanz, Laura I. Furlong, Olga Valverde

**Affiliations:** 1 Neurobiology of Behaviour Research Group (GReNeC-NeuroBio), Department of Experimental and Health Science, Universitat Pompeu Fabra, Carrer Dr Aiguader 88, 08003 Barcelona, Spain; 2 Neuroscience Research Program, IMIM-Hospital del Mar Research Institute, Barcelona, Spain; 3 Research Programme on Biomedical Informatics (GRIB), IMIM-Hospital del Mar Medical Research Institute, Universitat Pompeu Fabra, Barcelona, Spain; 4 MARGenomics core facility, IMIM-Hospital del Mar Medical Research Institute, Barcelona, Spain; 5 Department of Psychiatry, University of Oxford, Oxford, OX3 7JX UK; 6 Oxford Health NHS Foundation Trust, Oxford, OX3 7JX UK; 7 Johnson and Johnson Medical Ltd., Janssen-Cilag, High Wycombe, UK

**Keywords:** Alzheimer’s disease, Major depression, Gene Set Enrichment Analysis, Comorbidity, Transcriptome, Behaviour

## Abstract

**Background:**

Major depression (MD) is the most prevalent psychiatric disease in the population and is considered a prodromal stage of the Alzheimer’s disease (AD). Despite both diseases having a robust genetic component, the common transcriptomic signature remains unknown.

**Methods:**

We investigated the cognitive and emotional behavioural responses in 3- and 6-month-old APP/PSEN1-Tg mice, before β-amyloid plaques were detected. We studied the genetic and pathway deregulation in the prefrontal cortex, striatum, hippocampus and amygdala of mice at both ages, using transcriptomic and functional data analysis.

**Results:**

We found that depressive-like and anxiety-like behaviours, as well as memory impairments, are already present at 3-month-old APP/PSEN1-Tg mutant mice together with the deregulation of several genes, such as *Ciart*, *Grin3b*, *Nr1d1* and *Mc4r*, and other genes including components of the circadian rhythms, electron transport chain and neurotransmission in all brain areas. Extending these results to human data performing GSEA analysis using DisGeNET database, it provides translational support for common deregulated gene sets related to MD and AD.

**Conclusions:**

The present study sheds light on the shared genetic bases between MD and AD, based on a comprehensive characterization from the behavioural to transcriptomic level. These findings suggest that late MD could be an early manifestation of AD.

## Background

Depressive disorder affects over 4.4% of the population [1], and it is characterized not only by low mood but also by anhedonia, changes in appetite and sleep, feelings of worthlessness and guilt which may lead to attempts of suicide [1]. Major depression (MD) manifests throughout the life course including in older people when it is often associated with poor cognition that may not return to normality with effective treatment of the mood disorder [2, 3]. Recently, a genome-wide meta-analysis of depression reports 102 variants involving cell adhesion, dopaminergic and monoaminergic transmission, but also important gene–drug interactions that reveal the importance of antidepressant drug treating depression in prefrontal cortical regions of the brain [4]. Moreover, a genome-wide association study has identified 44 independent loci that define the genetic basis of MD in human patients, including synaptic genes, regulation of immune response and genes implicated in neuronal projection and neuron differentiation [5]. In addition to the cognitive phenotype of MD in older people, a substantial body of data strongly suggests the onset of MD in later life is associated with increased risk of Alzheimer’s disease (AD), the commonest form of dementia [6–8].

AD includes forgetfulness, memory disturbances and difficulty recognizing relatives and places [9]. AD is a neurodegenerative disease with a robust genetic component. Whereas late-onset AD shows a heritability of 58–79, early onset of AD shows over 90% [10]. Despite near 50 risk loci have been implicated in AD, such as amyloid precursor protein (*App*) and interleukin 34 (*Il-34*), among others [10], a wide body of literature provides compelling evidence of genetic pleiotropy for AD, having a quasi ‘monogenetic role’ of apolipoprotein E (*ApoE*) [11] because it confers a strong genetic risk for AD, cardiovascular and neurogenerative diseases [12, 13]. Although there is a controversy about if late-onset AD is generally considered a polygenetic disease [11], the early-onset AD pathology has a robust genetic component. Indeed, there is a close association between mutations and the number of APOE alleles, *App* and presenilin (*Psen*) genes with early-onset AD [14, 15], as observed in subjects with Down syndrome who carry three copies of APP and develop early-onset AD pathology, due to the trisomy of chromosome 21 [16]. Moreover, it has recently been described the implication of triggering receptor expressed on myeloid cells (TREM)-APOE pathway related with microglial β-amyloid plaque clearance [17] and neuroinflammatory responses in neurodegenerative diseases, such as AD [12], with a dysregulation of *Clec7* and *Itgax* transcripts related to inflammation and apoptotic processes, crucial components in the early-onset AD [18]. Furthermore, although cognitive and functional impairments are the predominant symptoms of AD, other behavioural and psychological symptoms of dementia (BPSD) that include depression, sleep and activity disturbances are common manifestations of the disease [19, 20]. These observations have led to the suggestion that MD may in some cases be a prodromal phase of AD. Indeed, both MD and AD have a considerable heritable component and changes in these systems have been shown in both disorders, including neuroinflammation [21, 22], oxidative stress [23] and certain dysregulations in cellular signalling [24] and neurotransmission [25, 26] among others. Indeed, recent studies have sought to uncover a common molecular signature between both AD and MD including from genome-wide association studies (GWAS) that have identified shared vulnerability genes for both disorders [27, 28].

Despite these advances, the aetiology of the comorbidity between AD and MD remains unknown although the observation that multiple behaviours that are reminiscent of BPSD are also observed in rodent models of disease [29], suggesting that there might have common molecular processes. Hereby, we study for the first time, using behavioural, transcriptomic and bioinformatic approaches, depressive-like symptoms in an AD mouse model (APP/PSEN1-Tg mice) at ages before the neuropathological features are manifest and when cognitive impairment is not yet evident [30]. To that end, we have performed RNA sequencing analyses to study changes that occur in brain areas related to the control of behavioural and cognitive behaviours, including the prefrontal cortex (PFC), striatum, hippocampus and amygdala, in mice at 3 and 6 months old. In order to study the mechanisms involved in the AD-MD comorbidity, a pre-ranked Gene Set Enrichment Analysis (GSEA) has been carried out. Using the transcriptomic results, we evaluated the enrichment in gene sets obtained from public resources containing functional and disease information.

## Material and methods

### Animals and rearing conditions

We used 30 hemizygous double transgenic male mice (B6C3-Tg (APPswe, PSEN1dD9)85Dbo/MmJax) model of AD (APP/PSEN1-Tg) and 30 male non-transgenic control mice (004462, The Jackson Laboratory, USA). Transgenic mice express a chimeric mouse/human APP (Mo/HuAPP695swe) and a mutant human presenilin-1 (PS1-dD9), each controlled by independent mouse prion protein promoter elements [31]. We employed APP/PSEN1-Tg and non-transgenic mice (*n* = 16 per group) at 3 months old and APP/PSEN1-Tg and non-transgenic mice (*n* = 14 per group) at 6 months old, before developing the β-amyloid plaques and right at onset [31]. All procedures were conducted in accordance with national and EU (Directive 2010-63EU) guidelines regulating animal research and were approved by the local ethics committee (CEEA-PRBB).

### Behavioural evaluation

#### Elevated plus maze (EPM)

Elevated plus maze (EPM) (Panlab s.l.u, Barcelona, Spain) was performed using a black maze elevated 30 cm above the ground [32]. The software SMART (Panlab s.l.u., Spain) automatically recorded the number of entries and the time spent in the arms.

#### Novel object recognition (NOR)

Single trial NOR was performed in an open black arena (32 × 28 cm) using 3 object types at opposite corners of the open field (OF), 50 mm from the walls, similar to those previously described [33]. The recognition index was defined as [*t*_Novel_/(*t*_Novel_ + *t*_Familial_)] × 100 for animals exploring novel objects in the acquisition trial.

#### Left–right discrimination learning

This test was performed using a T-maze apparatus, as previously described [34]. This T-maze was filled with water (23 ± 1 °C). During the first two trials, two identical platforms were submerged on the end of both arms to test possible side preferences. A mouse was considered to have achieved the criterion after 5 consecutive errorless trials. The reversal-learning phase was then conducted 48 h later, applying the same protocol except that mice were trained to reach the escape platform of the opposite arm. Escape latencies and number of trials to reach the criterion were manually recorded.

#### Tail suspension test (TST)

Each mouse was suspended individually 50 cm above a bench top for 6 min [32]. Mice were individually video-recorded and an observer, blind to the experimental conditions, evaluated the percentage of time the animal was immobile during the test.

#### Exposure to stress

The stressful procedure consisted in the exposition to two mild stressful situations each day during four consecutive days: animals were placed for 10 min in the OF apparatus, and then they were placed in glass cylinders filled with water during 6 min. Animals were subsequently evaluated for TST.

#### Habituation–dishabituation test

The test was performed as described [35]. After habituation to the cage, six consecutive 1-min presentations of a cotton swab with distilled water were followed by one presentation of limonene (Sigma-Aldrich) solution 1:1000 in distilled water. Tests were video-recorded, and a blinded observer measured the time that mice spent sniffing the cotton tip rearing on their hind limbs.

### Animal sacrifice and sample preparation for RNA extraction

Animals were sacrificed by cervical dislocation and brains were immediately removed from the skull. Brain samples were dissected at both ages: 3 and 6 months old from non-transgenic and APP/PSEN1-Tg mice (*n* = 6 per group). PFC, striatum, amygdala and hippocampus were dissected following an anatomical atlas [36], and they were immediately stored at − 80 °C until the RNA extraction. Then, each tissue sample was homogenized in 1 ml of QIAzol Lysis Reagent using a rotor–stator homogenizer (Polytron PT 2500 E; Kinematica AG, Switzerland) during 20–40 s. After homogenization, the RNA was extracted using RNeasy Lipid Tissue Mini Kit (Qiagen) [37].

### Library preparation and RNA sequencing

RNA samples (50–100 ng) with RIN scores from 7.6 to 9 (Agilent 4200 TapeStation) were reverse transcribed to cDNA. Poly-A tail selection was done using Total Dual RNASeq PolyA. cDNA was sequenced (HiSeq3000/4000) at the Oxford Wellcome Trust facility obtaining 75 bp per read. On average, 33M reads were obtained per sample and a mapping rate of ~ 85%.

### RNAseq DE analysis

Raw sequencing reads in the 1710 paired fastq files were mapped with STAR version 2.5.3a [38] to the GENCODE release 17 based on the GRCm38.p6 reference genome and using the corresponding GTF file. The 885 bam files corresponding to 9 different lanes were merged for each sample using Samtools 1.5, ending up with 95 bam files. The table of read counts was obtained with featureCounts tool in subread package, version 1.5.1.

Further analyses were performed in R, version 3.4.3. Genes having less than 10 counts in at least 10 samples were excluded from the analysis. Raw library size differences between samples were treated with the weighted ‘trimmed mean method’ TMM [39] implemented in the edgeR package [40]. These normalized counts were used in order to make the unsupervised analysis, heatmaps and clusters. For the differential expression (DE) analysis, read counts were converted to log2-counts-per-million (logCPM), the mean–variance relationship was modelled with precision weights using the voom approach and linear models were subsequently applied with limma package, version 3.30.13 [41]. *p*-values (*p*) were adjusted for multiple comparisons using the Benjamini–Hochberg false discovery rate (FDR) approach. Genes were considered to be differentially expressed if |logFC| > 0.585 and adjusted *p* < 0.05, where FC indicates fold change.

### qPCR validation

Data were obtained from the qPCR platform using Taqman low density array (TLDA; qPCR 7900HT, Life Technologies), where thirteen genes of interest were studied together with the following endogenous controls: *Gapdh*, *Tbp* and *18S*. To select the endogenous genes, we used the RNAseq data to study the stability across tissues and conditions of some genes obtained from Rojo and collaborators [42] and the TaqMan® Endogenous Control Assays. This stability was assessed in terms of mean and standard deviations. From the initial list of 7 potential endogenous genes (*Gapdh*, *Hsp90ab1*, *Tbp*, *Hprt*, *Gusb*, *Actb*, *B2m*), we selected the most stable ones, which are *Gapdh* and *Tbp*, and performed a geometric mean [43]. The gene *18S* was used to check overall expression whereas *Gapdh* and *Tbp* were geometric averaged and included in posterior analyses as the *C*_*t*_ *(endogenous)*. For each gene, DC_t_ was computed, comparing these values between conditions: *DC*_*t*_ *= C*_*t*_ *(gene) − C*_*t*_ *(endogenous)*. Comparisons between studied conditions were performed using a Student's *t*-test. Results were adjusted for multiple comparisons using the FDR. The comparisons performed in each area are APP/PSEN1-Tg and non-transgenic at both ages (validated genes in Supplementary Table 1).

### Functional and disease analysis

Pre-ranked GSEA was used in order to retrieve enriched gene sets corresponding to functional pathways [44]. The list of genes was ranked using the -log(p.val)*signFC value for each gene from the statistics obtained in the DE analysis with limma, as previously described. For the gene set collection, we used a database described previously [45] and available in http://ge-lab.org/gs. The gene sets in this database are harvested from different pathway data sources and published studies all related to mice. MGI gene IDs were converted to mouse symbol ID using the annotation files in www.informatics.jax.org. As there were a high number of redundant gene sets in the collection, a filtering strategy was applied to select the most representative ones (see Supplementary material). In brief, we performed a filtering step where (i) pairwise Jaccard coefficients were computed between the gene sets from the following data sources: published articles, MPO, HPO, Reactome, MsigDB, GO_BP, KEGG, PID, WikiPathways, INOH, PANTHER, NetPath, Biocarta, EHMN, MouseCyc and HumanCyc; (ii) for gene sets with Jaccard = 1 (gene sets constituted by the same genes), only one was considered; and (iii) from gene sets with Jaccard > 0.8, the gene sets with the highest number of genes was considered and other gene sets were excluded from the gene set collection.

#### DisGeNET database analysis

To evaluate the translation of our results in humans, we performed a GSEA to retrieve enriched gene sets associated with human diseases. The gene sets associated with human diseases, symptoms and traits were retrieved from the DisGeNET database [46] (https://www.disgenet.org/, v7). DisGeNET integrates gene–disease associations collected from curated repositories, from genome-wide association studies (GWAS) catalogues, from animal models, and data obtained from the scientific literature using text mining approaches [46]. For this analysis, we used only the curated gene sets in DisGeNET, which include information from repositories such as UniProt, PsyGeNET, Clingen and the Genomics England Panel app. The mouse genes were converted to human gene IDs using the annotation files in http://www.informatics.jax.org/. We ranked the genes using the -log(p.val)*signFC value from the DE analysis.

See Supplementary material and methods for additional details.

## Statistical analysis

For animal model experiments, analysis was performed using software SPSS 23.0 (SPSS Inc., USA). Statistical differences were investigated by Student’s *t*-test and ANOVA with repeated measurement analysis. *Post hoc* analysis was calculated with Bonferroni’s correction when applicable. Since habituation–dishabituation data were not normally distributed, Wilcoxon matched-pairs rank test and Kruskal–Wallis test were calculated. Fisher’s exact test was used to evaluate differences in the percentage of animals that made an error in the Y-maze. For the GSEA, we only included those gene sets with a FDR *q* value < 0.05 and |normalized enrichment score| > 1.4 (NES).

## Results

The transgenic mice expressing human APP/PSEN1-Tg carrying familial AD mutations used in these studies (B6C3-Tg) have an onset of plaque pathology at around 6 months [47], which is followed by cognitive impairments with both increasing by 12–18 months of age [48]. To explore behavioural alterations in these mice, we used a range of well-established experimental paradigms at 3 months (before the onset of pathology) and 6 months (at onset). Then, we evaluated the differentially expressed genes and pathways in the prefrontal cortex (PFC), striatum, hippocampus and amygdala at both ages.

### Anxiety-related behaviour in APP/PSEN1-Tg mice

**Fig. 1:**
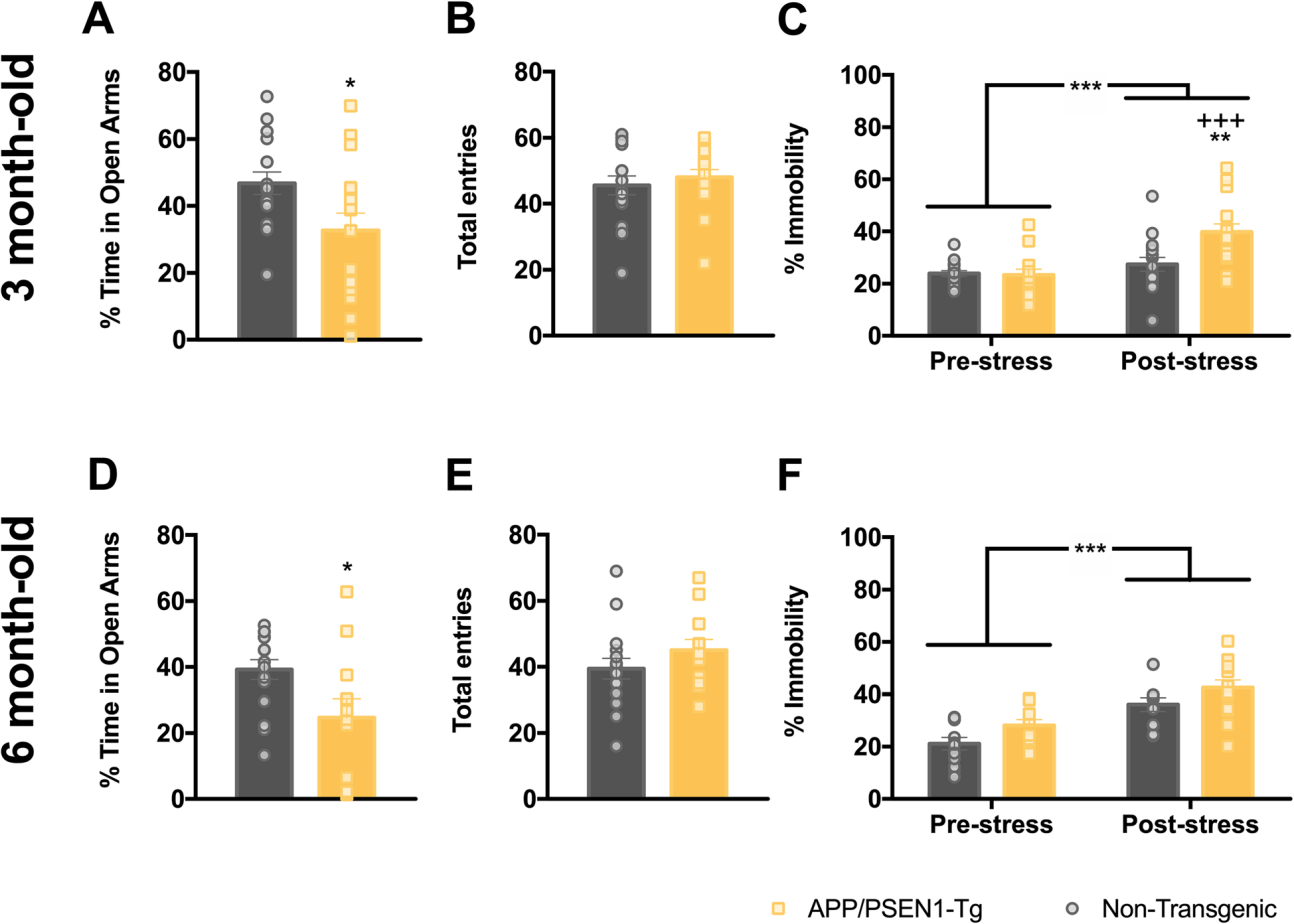
APP/PSEN1-Tg animals show early anxiety-like and despair-like behaviours induced by stress*.* Panels **a**, **b**, **d** and **e** represent the EPM results, and panels **c** and **f** correspond to the TST results. Grey (non-transgenic) and yellow (APP/PSEN1-Tg) bars represent the percentage of time spent in open arms (**a**, **d**) and total number of entries (**b**, **e**) at 3 (*n* = 16 per group) and 6 months (*n* = 14 per group) (*p < 0.05, Student’s *t*-test non-transgenic vs APP/PSEN1-Tg). Grey (non-transgenic) and yellow (APP/PSEN1-Tg) bars represent the percentage of time that animals are immobile in TST at 3 (*n* = 16 per group) (**c**) and 6 months old (non-transgenic *n* = 12, APP/PSEN1-Tg *n* = 14) (**f**) in pre-stress and post-stress conditions. Data are presented as mean ± SEM. ***p < 0.001 pre-stress vs post-stress condition; ^+++^*p* < 0.001 comparison of APP/PSEN1-Tg pre-stress vs post-stress; **p < 0.01 comparison of APP/PSEN1-Tg vs non-transgenic post-stress condition (two-way ANOVA)

To evaluate if APP/PSEN1-Tg animals experience anxiety-like behaviour, 3- and 6-month-old APP/PSEN1-Tg were subjected to EPM. The APP/PSEN1-Tg transgenic mice spent lower percentage of time in open arms than non-transgenic at both 3 (*t*_30_ = 2.30, *p* = 0.0286; Fig. 1a) and 6 months old (*t*_26_ = 2.419, *p* = 0.0229; Fig. 1d), whereas no differences were found in the total number of arm entries (*t*_30_ = 0.65, *p* = 0.520, Fig. 1b and *t*_26_ = 1.21, *p* = 0.236, Fig. 1e), indicating that APP/PSEN1-Tg animals displayed higher level of anxiety-like behaviours without locomotor impairments.

### Early onset of despair-like behaviour in APP/PSEN1-Tg mutant mice

Stress-induced immobility is a despair responsive phenotype often used as a proxy for mood state in rodent models. To evaluate this response, we performed the TST in APP/PSEN1-Tg transgenic and control mice. At 3 months old, the analysis of the TST results using an ANOVA for repeated measurements revealed *stress* (*F*_1,30_ = 20.77, *p* < 0.001), *genotype* (*F*_1,30_ = 5.27, *p* = 0.029) and *stress × genotype* (*F*_1,30_ = 8.61, *p* = 0.006; Fig. 1c) effects. After Bonferroni’s correction, results showed that transgenic mice presented higher stress-induced immobility (*p* < 0.001) in comparison with the pre-stress condition. Stress only increased the percentage of the immobility in APP/PSEN1-Tg mutants in comparison with non-transgenic animals (*p* = 0.005). At 6 months, the ANOVA for repeated measurements showed *stress* (*F*_1,24_ = 31.33, *p* < 0.001) and *genotype* (*F*_1,24_ = 4.62, *p* = 0.042; Fig. 1f) effects. These results indicate that transgenic mice spent a higher percentage of immobility time after repeated stress, but also this behaviour increases in both groups of mice.

### Early-adulthood long-term memory impairments in APP/PSEN1-Tg mice

**Fig. 2:**
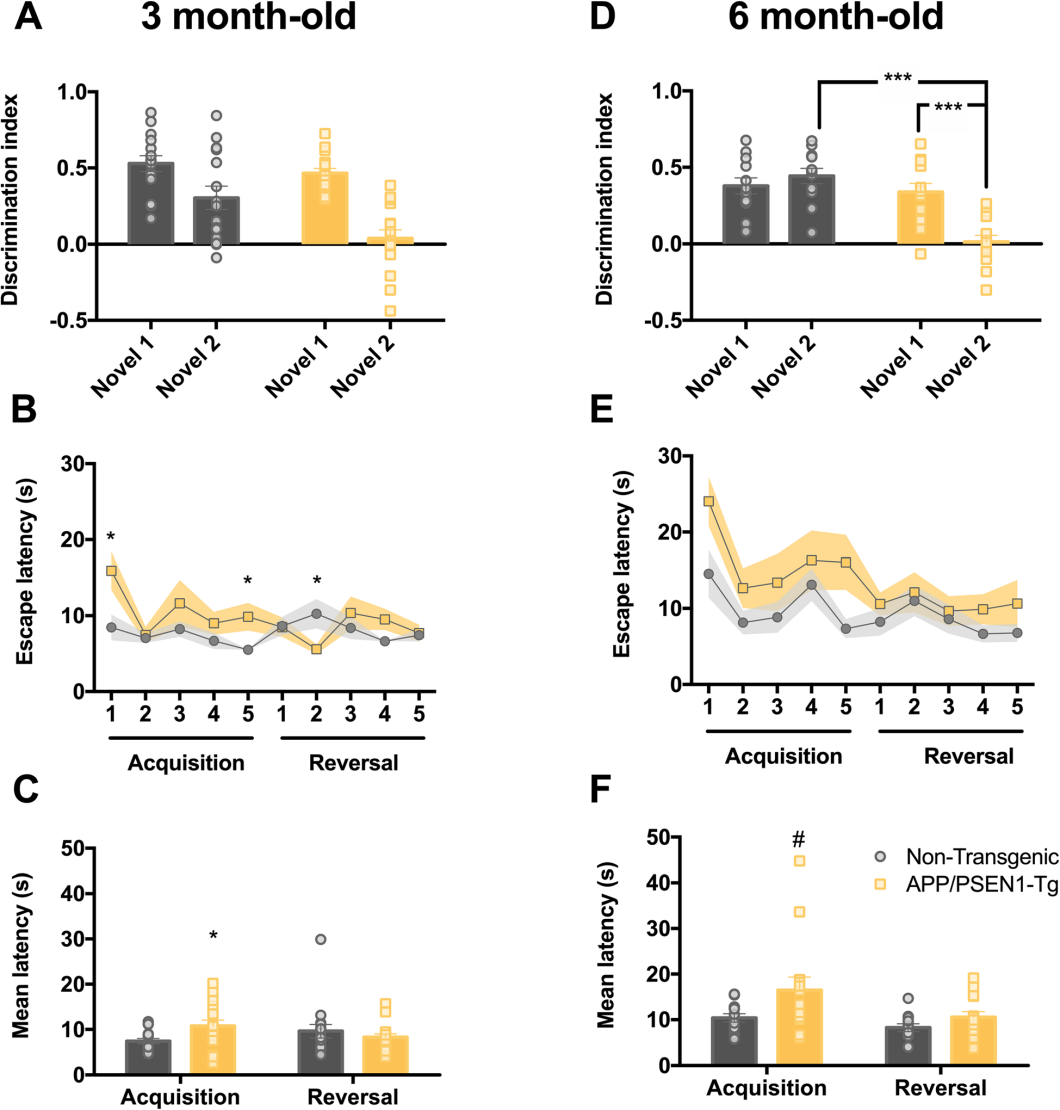
The onset of memory impairments in APP/PSEN1-Tg occurs at 3 months old*.* Novel object recognition (NOR) test: grey (non-transgenic) and yellow (APP/PSEN1-Tg) bars represent the novel object 1 and 2 discrimination index (%) at 3 (**a**) (non-transgenic *n* = 15, APP/PSEN1-Tg *n* = 16) and 6 months old (**d**) (non-transgenic *n* = 12, APP/PSEN1-Tg *n* = 14). Data are presented as mean ± SEM. *p < 0.05, **p < 0.01 (two-way ANOVA). T-maze left–right discrimination learning: **b**, **e** solid (non-transgenic) and dotted lines (APP/PSEN1-Tg) represent the escape latencies of both groups at two different ages (ANOVA of repeated measurements *p < 0.05; ^+^*p* < 0.05 comparison of APP/PSEN1-Tg vs the 1st trial; *p < 0.05 comparison of APP/PSEN1-Tg vs non-transgenic). **c**, **f** Grey (non-transgenic) and yellow (APP/PSEN1-Tg) bars represent the mean ± SEM trials to criterion during acquisition and reversal phase latencies at 3 (*n* = 16 per group) and 6 months old (non-transgenic, *n* = 12, APP/PSEN1-Tg *n* = 14). **f** At 6 months old, transgenic mice tend to reach the criterion later than control groups during acquisition (Student’s *t*-test, non-transgenic vs APP/PSEN1-Tg, *p < 0.05; ^#^*p* = 0.068)

Loss of memory is affected early during the time course of the AD, being one of the most recognizable symptoms of the disease [49]. We assessed the short- and long-term recognition memory in 3- and 6-month-old APP/PSEN1-Tg mice using the NOR test. At 3 months old, a two-way ANOVA showed a *genotype* (*F*_1,29_ = 8.30, *p* = 0.007) and *object* effect (*F*_1,29_ = 36.93, *p* = 0.001; Fig. 2a). The *genotype* effect indicates that control animals had greater discrimination index than APP/PSEN1-Tg group. At 6 months, a two-way ANOVA revealed a main effect of *genotype* (*F*_1,24_ = 22.44, *p* < 0.001), an *object* effect (*F*_1,24_ = 6.51, *p* = 0.018) and *object × genotype* interaction (*F*_1,24_ = 14.64, *p* = 0.001; Fig. 2d). The *genotype* effect shows that control animals had greater discrimination index than transgenic mice. The *post hoc* Bonferroni’s analysis showed that non-transgenic mice could discriminate both the novel object 1 and 2 in the same manner (*p* = 0.394), whereas APP/PSEN1-Tg animals could only discriminate novel object 1, having a greater discrimination index for the novel object 1 than novel object 2 (*p* < 0.001). Non-transgenic animals had a higher discrimination index than APP/PSEN1 when they are exposed to novel object 2 (*p* < 0.001). In fact, APP/PSEN1-Tg animals could not discriminate between familial and novel object 2, indicating important memory impairments.

### Early poor left–right discrimination learning of APP/PSEN1-Tg mice

In order to evaluate working memory and attention deficits present in AD [50], we performed a left–right discrimination learning paradigm in 3- and 6-month APP/PSEN1-Tg transgenic and non-transgenic mice using the T-maze. The two-way ANOVA showed a *trial* effect (*F*_9,270_ = 2.152, *p* = 0.026) and *trial × genotype* (*F*_9,270_ = 2.023 *p* = 0.037; Fig. 2b) at 3 months old. The *post hoc* analysis revealed that APP/PSEN1-Tg animals took more time to reach the platform than control animals on trial 1 (*p* = 0.044) and trial 5 (*p* = 0.027) during the acquisition learning. However, control mice spent more time to reach the platform during trial 2 of the reversal-learning phase (*p* = 0.02) than transgenic mice. The pairwise comparison showed that APP/PSEN1-Tg reduced the time to escape on acquisition trial 2 (*p* = 0.037) and trial 2 of the reversal learning (*p* = 0.017) in comparison with trial 1 of the acquisition. We found that APP/PSEN1-Tg animals spent more trials than non-transgenic animals (*t*_30_ = 2.35, *p* = 0.026; Fig. 2c) to reach the acquisition criterion. Nevertheless, both groups spent a similar number of trials to reach the criteria during reversal learning (*p* = 0.443). At 6 months old, the ANOVA revealed a *trial* (*F*_9,216_ = 3.84, *p* < 0.001) and *genotype* (*F*_1,24_ = 6.53, *p* = 0.017, Fig. 2e) effects, suggesting that APP/PSEN1-Tg mutant mice showed longer latencies to reach the platform than control animals during acquisition and reversal-learning phases. The mean latency to get the acquisition criteria showed a trend to be statistically different in the number of trials that APP/PSEN1-Tg in comparison with control mice (*t*_24_ = 1.91; *p* = 0.068; Fig. 2f).

### Hyposmia in APP/PSEN1-Tg mice at 6 months old

**Fig. 3:**
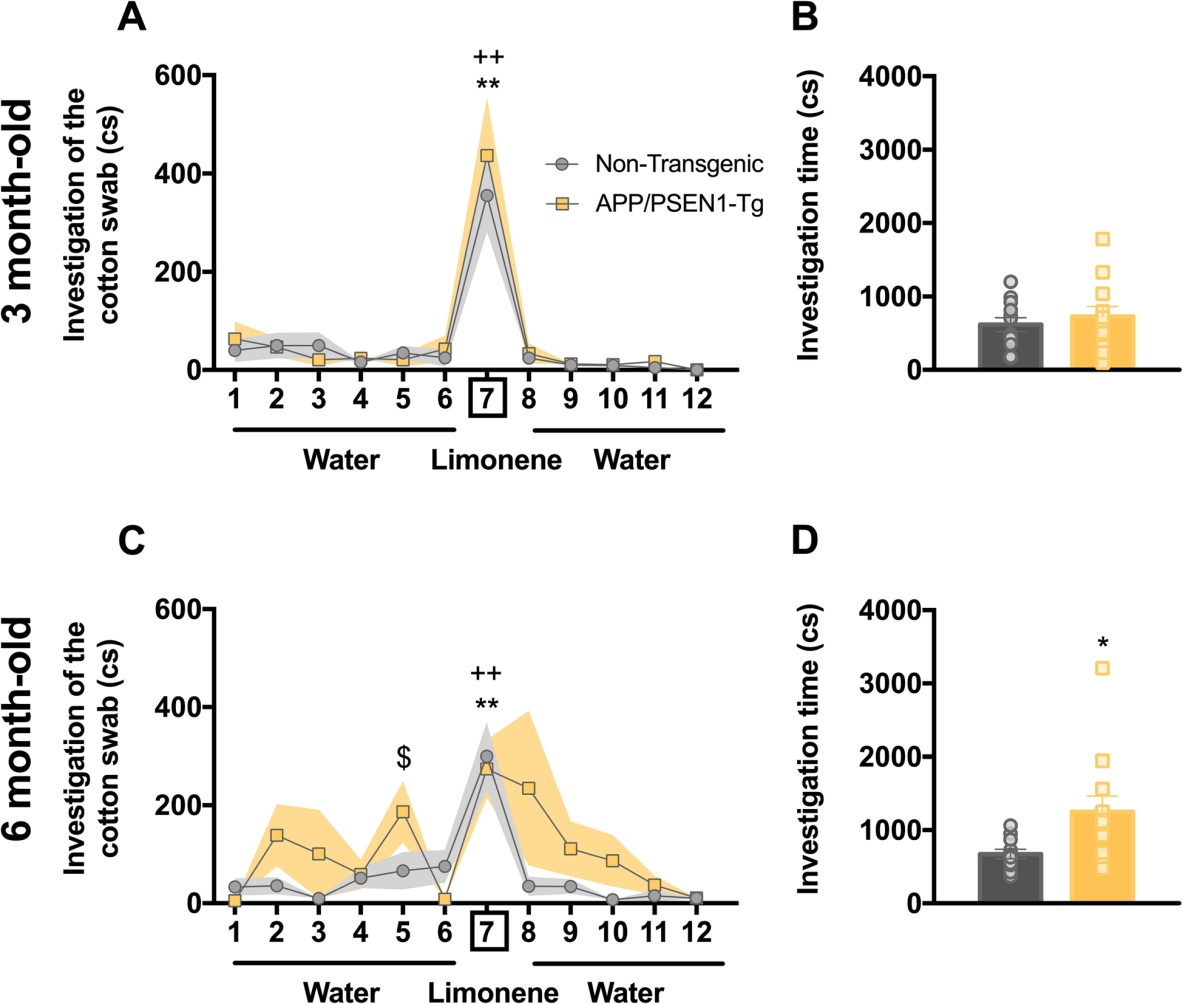
APP/PSEN1-Tg mice show olfaction disruptions at 6 months old. Graphs show the time (centiseconds, cs) that APP/PSEN1-Tg and non-transgenic animals spent investigating a cotton swab through consecutive 1-min presentations (mean ± SEM) of distilled water (presentations 1–6 and 8–12) and limonene (presentation 7) at 3 (**a**) and 6 months (**c**). The exploration time during the first presentation of the cotton swab with water [1] was compared with that of the first presentation of limonene [7] in each group (Wilcoxon test, **p < 0.01 comparison of APP/PSEN1-Tg water vs limonene presentations; ^++^*p* < 0.01 comparison of non-transgenic water vs limonene presentations; Kruskal–Wallis test ^$^*p* < 0.05 comparison of APP/PSEN1-Tg vs Non-Transgenic). Grey (non-transgenic) and yellow (APP/PSEN1-Tg) bars represent the total investigation time at 3 (**b**) (*n* = 12 per group) and 6 (**d**) months old (non-transgenic *n* = 11, APP/PSEN1-Tg *n* = 12) (Student’s *t*-test *p < 0.05). Data are presented as mean ± SEM

Since olfactory impairment is present in up to 90% of AD patients [51], we assessed the olfactory function in transgenic and control mice (Fig. 3a, c). At 3 months old, both experimental groups could discriminate between the first water presentation and limonene odour (Wilcoxon test, non-transgenic group, *p* = 0.05 and APP/PSEN1-Tg, *p* = 0.01; Fig. 3a). At 6 months, both APP/PSEN1-Tg transgenic mice and control mice could discriminate between the first water presentation and limonene (Wilcoxon test, non-transgenic, *p* = 0.006, and APP/PSEN1-Tg, *p* = 0.008; Fig. 3c). Additionally, APP/PSEN1-Tg mutant mice showed a higher investigation time during the fifth water presentation than the control group (Kruskal–Wallis test, *χ*^2^ = 3.84, *p* = 0.05; Fig. 3c). Whereas both experimental groups spent the same time investigating the cotton swab at 3 months old (*t*_22_ = 0.66, *p* = 0.52; Fig. 3b), APP/PSEN1-Tg increased the investigation time than non-transgenic control animals, showing an indiscriminate investigation at 6 months old (*t*_21_ = 2.52, *p* = 0.02; Fig. 3d), independently of the type of odour presented.

### DE analysis in the PFC, striatum, hippocampus and amygdala from mice at 3 and 6 months old

**Fig. 4:**
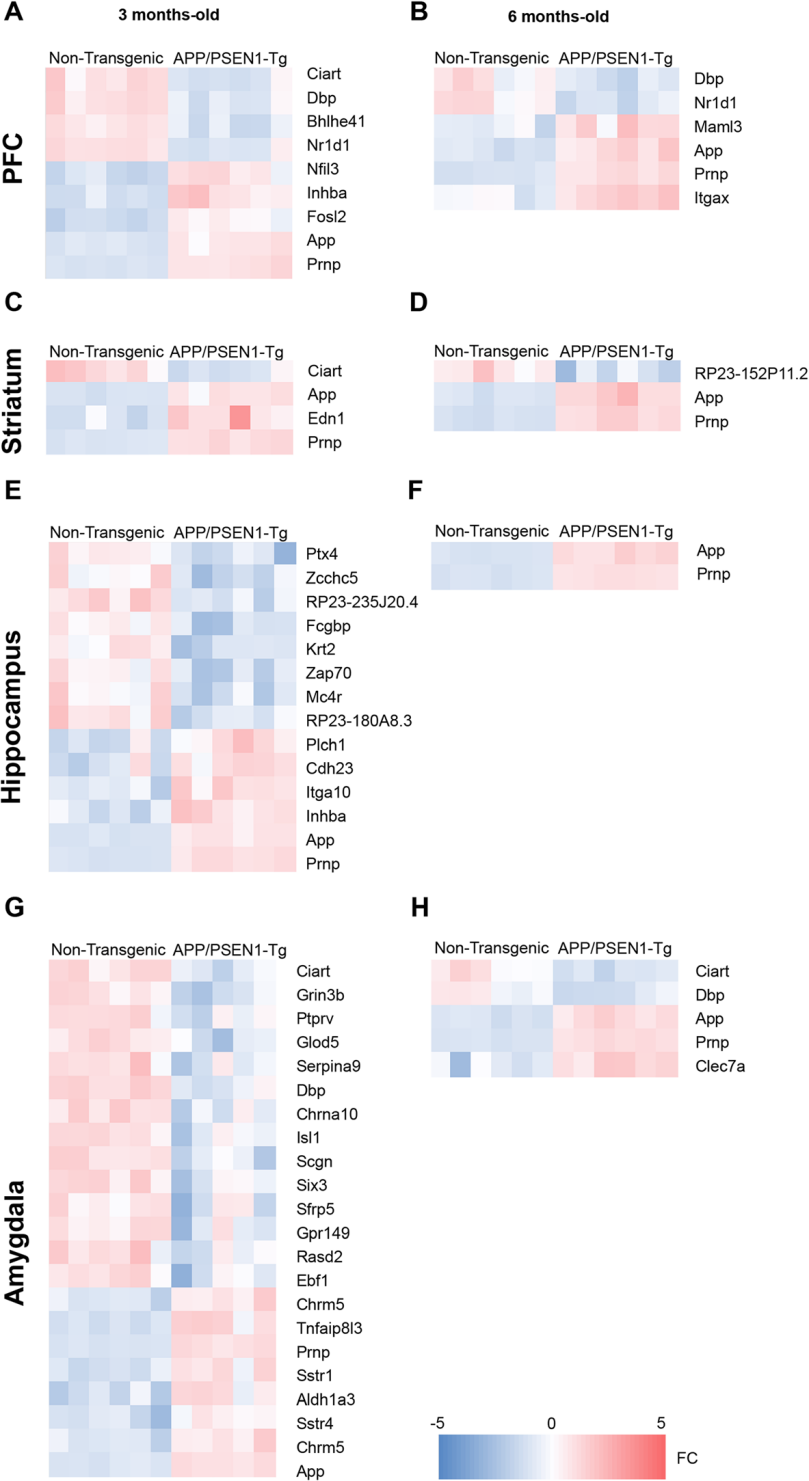
Differentially expressed genes in the PFC, striatum, hippocampus and amygdala from APP/PSEN1-Tg mice at 3 and 6 months old. Heatmaps representing the degree of change for the differentially expressed genes at 3 (**a**, **c**, **e**, **g**) and 6 (**b**, **d**, **f**, **h**) months between control and APP/PSEN1-Tg (5–6 independent brain samples per area). **g** The heatmap from the amygdala at 3 months old is a reduced representation from > 100 up- and downregulated genes (*p* < 0.05;|logFC| > 0.59). Legend (bottom) indicates the colour-coded fold-change scale (− 5 < FC < 5) where negative values represent downregulation in blue, and positive upregulation in red

We performed gene expression analysis in regions of the brain known to be loci for these functions. We compared the transcriptome signature of APP/PSEN1-Tg and control mice at 3 and 6 months old, to determine the significant differentially expressed (DE) genes with a cut-off |logFC | < 0.585, adjusted *p* < 0.05, deregulated in the brain areas of interest (Fig. 4; Supplementary Tables 2, 3, 4 and 5).

Among the dysregulated genes in PFC of younger APP/PSEN1-Tg mice, we found 5 corresponding to the so-called canonical clock genes: *Ciart*, *Dbp*, *Bhleh41* and *Nr1d1* were downregulated, and *Nfil3* was upregulated, together with other genes, such as *App*, *Prpn*, *Inhba* and *Fosl2* (Fig. 4a; Supplementary Table 2). At 6-month-old mice, *Dbp* and *Nr1d1* remained downregulated in this area, whereas *App* and *Prnp* were also upregulated together with *Itgax* and *Maml3* (Fig. 4b; Supplementary Table 2).

In the striatum of 3-month-old mice, we only found the downregulation of *Ciart*, and the overexpression of *App*, *Prpn* and *Edn1* (Fig. 4c; Supplementary Table 3). By contrast, the deregulation of older mice in the striatum was restricted to only one downregulated transcript, *RP23-152P11.2*, while *App* and *Prpn* remained upregulated (Fig. 4d; Supplementary Table 3).

In the hippocampus of 3-month-old APP/PSEN1-Tg mice, 8 genes were downregulated, including *Ptx4* and *Mc4r* and *Zap70*. Among the upregulated genes, we found *Plch1*, *Cdh23*, *Itga10*, *App*, *Prnp* and *Inhba* (Fig. 4e; Supplementary Table 4). At 6 months, the analysis revealed that only *App* and *Prnp* remained upregulated (Fig. 4f; Supplementary Table 4).

**Fig. 5:**
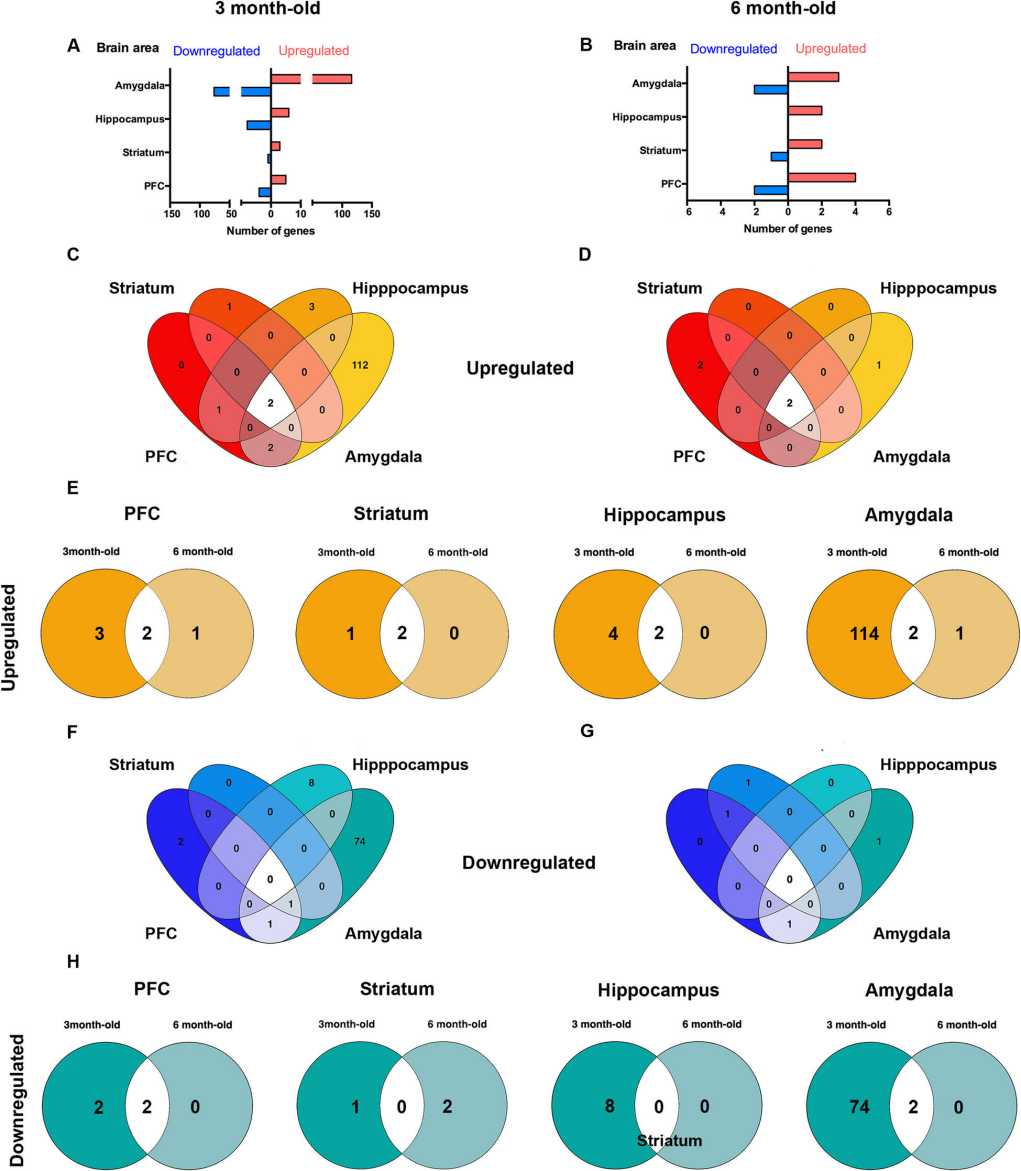
Distribution pattern of differentially expressed genes from the APP/PSEN1-Tg mouse model. Total number of up- (red bars) and downregulated (blue bars) genes within the amygdala, hippocampus, striatum and PFC at **a** 3 months and **b** 6 months. Venn diagrams show the number of differentially expressed genes among areas, in an upregulated (**c**, **d**) and downregulated (**f**, **g**) way. Venn diagrams show the number of upregulated (**e**) and downregulated (**h**) shared genes between 3 and 6 months old of age APP/PSEN1-Tg (*n* = 6 per group) vs non-transgenic mice (*n* = 6 per group)

Interestingly, the amygdala of young transgenic mice was the most affected structure. More than 100 genes were upregulated in the amygdala of the 3-month-old AD mice (Fig. 5a; Supplementary Table 5), including *Tnfαip8l3*, *Sstr1*, *Sstr4*, *Chrm5* and *Aldh1a3* (Figs. 4g; 5a, c). Among the almost 100 downregulated genes in the amygdala at 3 months old (Fig. 4g; Supplementary Table 5), we found *Grin3b*, C*hrna10* and *Ciart*. However, when we analysed the number of deregulated genes at 6 months old, both the upregulated and downregulated genes dramatically decreased in all brain areas (Figs. 4h, 5b). In the amygdala, the upregulation of genes diminished to three genes, *App*, *Prnp* and *Clec7a* (Figs. 4h, 5b, d) while the downregulated genes were restricted to *Dbp* and *Ciart* (Figs. 4h; 5c, h).

When we compared those genes that were consistently deregulated at both ages per area, we observed that the upregulation of *App* and *Prnp* were extended throughout PFC, hippocampus and amygdala (Figs. 4a, e, g; 5e). Moreover, this upregulation at 6 months old was affecting all brain regions (Figs. 4b, d, f, h; 5e). By contrast, *Ciart* was downregulated in the PFC, striatum and amygdala of APP/PSEN1-Tg mice at 3 months old. Only *Nr1d1* and *Dbp* in PFC and *Dbp* and *Ciart* in the amygdala were downregulated in the older transgenic mice (Figs. 4b, h; 5h).

### Enriched gene sets at 3 and 6 months old

**Fig. 6:**
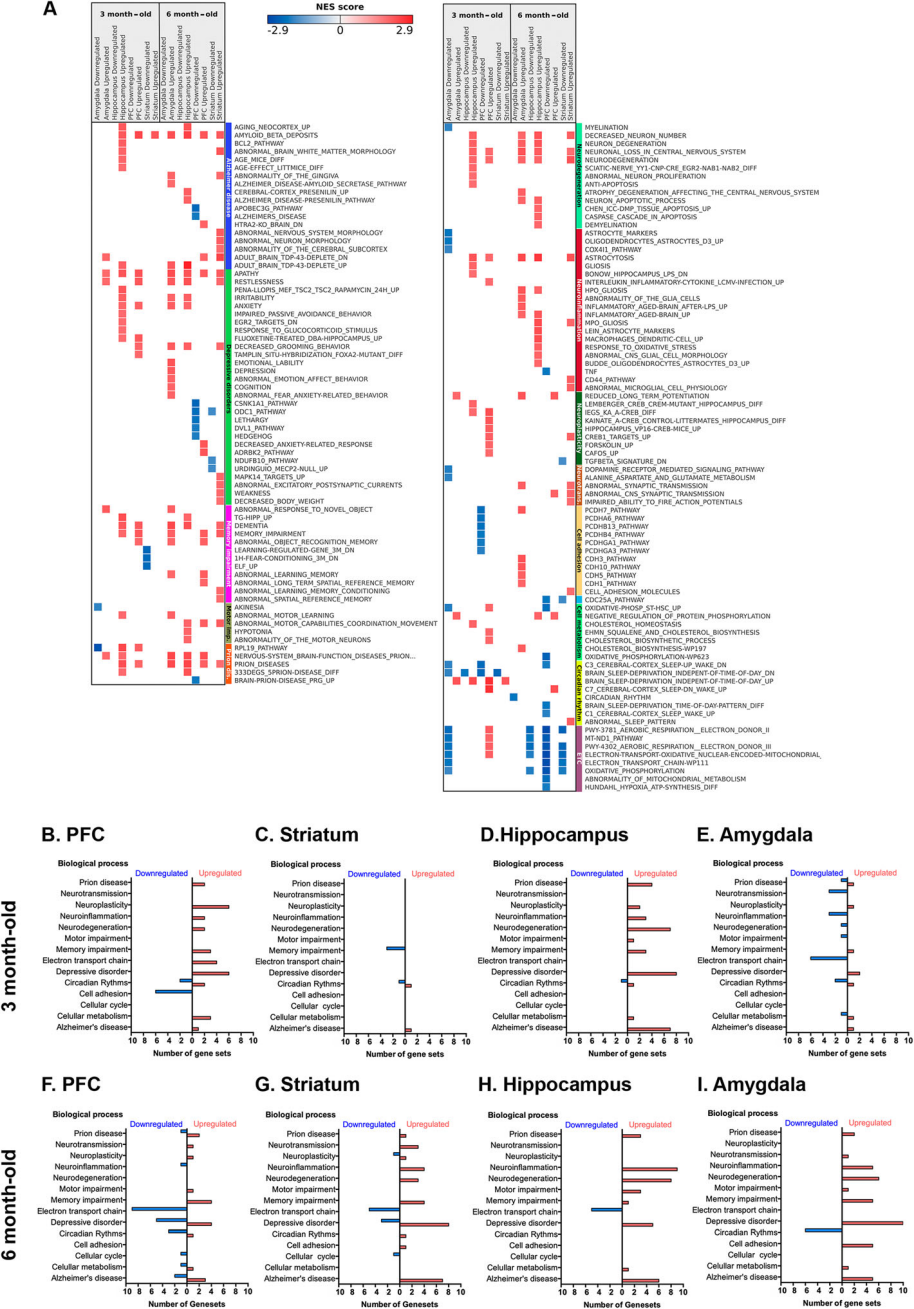
Comparison of differentially expressed gene sets in APP/PSEN1-Tg mice in the PFC, striatum, hippocampus and amygdala. **a** The heatmap represents some selected up- and downregulated gene sets at 3 and 6 months old included in different biological processes: Alzheimer disease, depressive disorders, memory impairment, prion disease, motor impairment (Motor imp.), neurodegeneration, neuroinflammation, neuroplasticity, neurotransmission (Neurotrans.), cell adhesion, circadian rhythm and electron transport chain (ETC) (FDR *q* value < 0.05; |normalized enrichment score| > 1.4.). List of the total number of differentially expressed gene sets ordered by biological processes found in the PFC (**b**, **f**), striatum (**c**, **g**), hippocampus (**d**, **g**) and amygdala (**e**, **j**) at 3 and 6 months old, respectively. Red bars (positive) represent the upregulated expressed gene sets and the blue bars (negative) show downregulated gene sets in each area. For the GSEA, we only included those gene sets with FDR *q* value < 0.05 and |NES| > 1.4

We then performed a GSEA to gain insight on the function of the genes differentially expressed in transgenic mice in the pre-disease and early pathology periods (3 months and 6 months), using information from pathway and disease databases. This functional analysis showed that enriched gene sets related to depressive disorders appear at 3 months old within the PFC, hippocampus and amygdala, areas that are intimately involved in cognitive function and mood regulation (Fig. 6a, b, d, e; Supplementary Table 6). In the striatum, we identified two positively enriched gene sets—those of circadian rhythms and Alzheimer’s disease (Fig. 6c)—that also appear in the PFC, hippocampus and amygdala at early stages. In 6-month-old animals, however, the number of gene sets that were significantly enriched in APP/PSEN1-Tg transgenic mice was higher. We identified additional enriched pathways linked to the electron transport chain, found in the PFC, striatum and hippocampus (Fig. 6f, g, h; Supplementary Table 7) and pathways implicated in AD and neuroinflammation. In parallel, the positively enriched gene sets related to depression were observed in all the brain structures analysed (Fig. 6f–i) at this later age. Additionally, the neurotransmission-related pathways were notably enriched in 6-month-old transgenic mice, suggesting that neurodegeneration increased the number of affected gene sets. Moreover, the impairments in circadian rhythms appeared in the preclinical phase in the amygdala (Fig. 6b–e), persisting when pathology becomes apparent (Fig. 6i).

### DisGeNET GSEA supports early-cognitive disabilities in 3-month-old animals

To investigate if the genes that were found to be perturbed in the animal model have been also associated with human diseases, and thus, to assess the translational potential of our results, we then performed a GSEA analysis using DisGeNET (version v7.0; http://www.disgenet.org/) gene sets (Additional file 1: Supplementary Figure S3; Supplementary Tables 8 and 9). This GSEA, which is specific for diseases and disease-related phenotypes, showed positively enriched gene sets corresponding to several subtypes of AD and dementias in the PFC and hippocampus of 3-month-old animals. We found positively enriched gene sets associated with memory impairment phenotypes, anxiety disorders, traits and symptoms related to motor impairment, and to neurodegenerative diseases (Additional file 1: Supplementary Figure S3) in the hippocampus of younger animals. These results are consistent with the deregulated pathways shown in Fig. 6 and with the behavioural results. In 6-month-old animals, the DisGeNET GSEA showed that positively enriched gene sets of several AD subtypes and dementias, and those related to memory impairment were extended to all brain areas. This dysregulation to memory-related impairment in old animals is consistent with working memory deficits and learning impairments that displayed 6-month-old animals performed in the behavioural experiments. Moreover, positively enriched gene sets related to cerebrovascular diseases, which have been related to MD [52], were observed in the hippocampus and striatum of 6-month-old animals. Most of these disorders are mediated by inflammation in MD [53], which is consistent with the dysregulation of neuroinflammation pathways found in the hippocampus of young and old animals (Fig. 6a, d, h).

## Discussion

This study provides the first evidence of early, pre- and peri-pathological depression-like and anxiety-like symptoms, cognitive impairments and the underpinning transcriptomic changes specifically in the PFC, striatum, hippocampus and amygdala in a rodent AD model (B6C3-Tg (APPswe, PSEN1dD9)85Dbo/MmJax). In this mouse model, the anxiety-related behaviours and despair-like behaviours after stressful conditions, hyperlocomotion (Additional file 1: Supplementary Figure S1A and D) and diminishing discrimination begin at 3 months of age, before the onset of AD pathology. Moreover, the hyposmia traits, working memory deficits and reversal-learning impairments appear later at 6 months old, at the time when amyloid plaque pathology begins to become apparent. Most of these alterations observed in APP/PSEN1-Tg mice have a global correspondence in humans before clinical manifestations of AD. In fact, current research of this pathophysiology is critical to detect incipient symptomatology of AD patients (Table 1), playing a crucial role to identify preclinical individuals. Notably, patients in prodromal phases of AD show memory impairments [59, 62], depression [6, 54, 55], anxiety [55] and reduction of olfaction function [61], as we observed in APP/PSEN1-Tg mice at 3 and 6 months of age. Although we could not forget the presence of other biomarkers that are more frequently observed in early onset of AD in humans, such as irritability [55, 56] and apathy/indifference [56], they are not easily measurable in rodent models.

**Table 1:** Behavioural results in APP/PSEN1-Tg model recapitulates most of the symptomatology in prodromal stages of human AD. In every square, we observe some of the alterations (blue) in human subjects before the AD diagnosis, and the respective behavioural results in APP/PSEN1-Tg mouse model (white) before the massive amyloid deposition obtained in the present study. ↑ increased; ↓ decreased; = unaltered

Alterations	Mouse in the present study		Human
	3 months old	6 months old	Previous to AD
Anxiety			↑ [54–56]
Elevated plus maze	↑ Anxiety-related behaviours	↑ Anxiety-related behaviours	
Open field	↑ Anxiety-related behaviours	↑ Anxiety-related behaviours	
Working memory			↓ Working memory ↓ Attention in patients with mild cognitive impairment before the AD diagnosis [57]
Spontaneous alternations in Y-maze	=	↓	
Recognition memory			↓ Recognition discriminability as a prodromal AD [58]
Short-term memory (NOR)	=	=	
Long-term memory (NOR)	↓	↓	
Spatial memory			↓ Spatial navigation in preclinical AD cases [59]
Left–right discrimination learning	↓	↓	
Depression			↑ [54, 55]
Tail suspension test	↑ Despair-like behaviour only after stress	↑ Despair-like behaviour	
Locomotion			↓ Walking speed in APOE4 carrier participants with mild cognitive impairment [60]
Locomotor activity	↑	↑	
Olfactory discrimination			↓ Olfactory function before clinical manifestations of AD [61]
Habituation–dishabituation test	=	↓ (hyposmia)	

The behavioural experiments indicate that APP/PSEN1-Tg mice showed early anxiety-like (Fig. 1a, d; Additional file 1: Figure S2) and depressive-like traits (Fig. 1c, f), symptoms which would be expected to be associated with dysfunction of the amygdaloid, hippocampal and PFC circuitries. Supporting these observations, the genes *Nr1d1* and *Bhlhe41*, which have been associated with the control of circadian rhythm function and depression [63, 64], were downregulated in the PFC of transgenic mice. Moreover, *Plch1* is upregulated at 3 months old in the transgenic mice. The product of this gene is part of the pathway generating second messengers, such as inositol 1,4,5-triphosphate and diacylglycerol, a pathway which is intimately linked to the depressive-like phenotype [65, 66]. At 3 but not at 6 months old, we found the upregulation of *Inhba* in the PFC and hippocampus, which could represent a compensatory change to mitigate the depressive-like behaviour. In fact, the activin signalling pathway plays a key role in the response to antidepressant treatment in humans [67]. *Nr1d1* remains downregulated at 6 months old, without changes in *Inhba* signalling, due to possible deeper detrimental effects at older ages, e.g. deficits in working memory. Additionally, *Mc4r* is downregulated in the hippocampus at 3 months old and facilitates anxiety- and depression-like behaviours pursuant to chronic stress [68]. Indeed, a general overexpression of *Prnp* and *App* in 3- and 6-month-old APP/PSEN1-Tg mutant mice could also contribute to cognitive decline, since previous studies found alterations of these proteins in cortical areas and peripheral blood of MD [69, 70]. Additionally, the overexpression of *Prnp* is also associated with increased anxiety levels in mice [71]. Noteworthy, this mouse overexpresses APP695 containing the double Swedish mutation, so the general upregulation of *App* found in all brain areas of interest is directly induced by the model. Moreover, *Prnp* may modulate depressive-like behaviour in mice via interactions with monoaminergic neurotransmission [72].

In accordance, we observed an increasing number of enriched pathways linked to mood disorders (including depression, apathy and anxiety, among others) and disease-associated gene sets at 3 and 6 months old, supported by DisGeNET GSEA (Additional file 1: Figure S3), before the period of formation of β-amyloid plaques in this model [31]. It should be noted that apathy, depression and anxiety are neuropsychiatric symptoms related to increased risk factors for dementia and mild cognitive impairment that could represent early manifestation of AD [73]. DisGeNET GSEA showed some anxiety traits as disease-associated gene sets at 3-month-old mice, whereas depressive and anxiety disorder gene sets were revealed at 6 months old, supporting behavioural results. Thus, the emotional alterations observed in this rodent model could be an early manifestation of the symptomatology-like of AD, as hypothesized for human disease. Samples from two studies of ageing such as the Religious order Study and Memory and Aging Project (ROSMAP) [74] would be helpful since it recruits individuals without dementia and provides information about not only histological post-mortem information but also transcriptional [75] data, neuroimaging and characterization of the subjects previous to death. Although the comparison between transcriptomic information of these prospective studies and our animal model results would result in an interesting point of view, it should be taken with caution because these human samples correspond to older individuals, when AD was diagnosed. In this sense, there are few studies that could provide comparable information with our transcriptomic results. BIOCARD team studies are aimed at identifying biomarkers of early cognitive impairment and dementia, with a particular focus on AD, by collecting data on cognitive testing, magnetic resonance imaging and blood specimens [76, 77] using younger subjects. Recently, the authors have recently described that low-severity depressive symptomatology is related to an increased risk of progression to clinical AD in patients diagnosed with mild cognitive impairment [78].

Overall, the APP/PSEN1-Tg mutant mice had a general decline in memory with ageing [29, 34] but this phenomenon appeared before the onset of pathology in our mouse model. Despite a subtle imbalance of memory impairment pathways at 3 months old, transgenic mice showed higher latencies to acquire the criterion in the left–right discrimination test. However, working memory analysed in the Y-maze test remained unaltered at 3 months old (Additional file 1: Figure S1B). The memory deficits could be promoted by the observed dysregulation of *Cdh23*, *Ptx4* and *Mc4r* genes in the hippocampus, as they are known to participate in the endocytosis of glutamatergic ionotropic AMPA receptors, hippocampal synaptic plasticity and mood-like disorders in mice [79–82]. At 6 months old, APP/PSEN1-Tg mice show deeper deficits in memory in all the evaluated tasks. Transgenic animals showed a poor left–right discrimination, long-term memory deficits in NOR test and lower percentage of alternations evaluated in the Y-maze test. Moreover, a higher percentage of animals performed some error during working memory task, indicating a progressive working memory decline with age (Additional file 1: Figure S1E and F). These memory impairments run in parallel with an increasing number enriched pathways in the PFC and hippocampus (abnormal long-term spatial reference and spatial memories, among others), involved in working [83, 84] and spatial learning [85, 86], respectively, and with dysregulation of gene sets related to memory impairment and learning disabilities as DisGeNET GSEA support (Additional file 1: Figure S3).

Our results show that the APP/PSEN1-Tg, at 3 months old, mice spent the same time as the control mice investigating the limonene impregnated cotton swabs. However, at 6 months old, the exploration of both odours was increased in transgenic mice compared to the control group, independently whether water and limonene were presented. Overall, APP/PSEN1-Tg animals seem not to present a complete anosmia, although it seems that they display a loss of discrimination between odours, so this possible hyposmia together with working memory (Additional file 1: Figure S1E and F) [83, 84] impairments could explain the indiscriminate investigation that transgenic mice display. These olfactory alterations could be due to pathology in the olfactory bulb at 6 months old [87], which might be promoting changes in the sense of smell of APP/PSEN1-Tg mice. Thus, our mouse model could reflect the progressive anosmia that AD patients show at early stages of the disease [51]. Indeed, a recent study shows that late-depression patients show similar olfactory impairments than those in AD [88]. Thus, early olfactory deficits could lead to the identification of depression patients with high risk of developing AD [88], as also occurs in our APP/PSEN1-Tg animals by showing early depression-like behaviour with later hyposmia traits.

MD and AD are both associated with neuroinflammation and oxidative stress [23], and evidence for the activation of both processes was observed in the differential expression analysis. This shows a dysregulation of inflammatory-related response genes, such as *Maml3*, *Zap70*, *Itgax* and *App* [12, 89–92] at both ages in APP/PSEN1-Tg mice. However, it should be noticed that there are important immunological differences between the human and mouse immune systems, making the direct comparison more difficult. These discrepancies should be taken into account when using mice as preclinical models of human disease. Despite most of the genes showing similar expression of individual pairs of orthologous genes between human and mouse [93], there are important differences in the innate and adaptative responses between both species [94]. It has been identified at least 169 divergent orthologous genes related to cell surface markers and cytokines that differ between human and mouse that were not previously reported [93]. In addition, gene sets related to electron transport chain are, in general, downregulated in APP/PSEN1-Tg animals at 6 months in the PFC, striatum and hippocampus, reflecting a possible abnormal function of mitochondrial metabolism, as previously observed in AD [95] and MD [96].

One striking observation is that the majority of the negatively enriched gene sets are associated with control of circadian rhythms, and these are observed in all brain areas of interest (Fig. 6b). In this sense, our results support previous research studies that examined the presence of circadian dysfunction in AD [97, 98] and MD [99, 100]. We observe the downregulation of *Ciart* and *Dbp* in the amygdala, striatum and PFC at 3 and 6 months old (Fig. 5 for more details). The expression of both genes in the anterior cingulate cortex modulates the circadian rhythms and mood [64, 99], through the MERK/ERK signalling pathway. Although disturbances in the circadian rhythms are associated with MD and AD [101], this is the first study in which both circadian transcripts are linked to AD. A close relationship has been found between *Dbp* and AD, given that TGF-β2, present in the cerebrospinal fluid of AD patients, inhibits the expression of some clock genes, including *Dbp*, in in vitro assays. We also note that the predominant tau-kinase, reported to be downstream of β-amyloid production [102–104], GSK3β, is a regulator of Per2 phosphorylation and hence a master-regulator of the mammalian clock [105]. Our results suggest that there is a decline in the functional control of circadian rhythms in APP/PSEN1-Tg mice that should be further studied in detail.

## Strengths and limitations

The strengths of this study include a well-characterization of cognitive and emotional deficits at peri-pathological stages in a murine AD model, a wide transcriptomic analysis comparing four different brain areas at two different ages, and a translational support for common deregulated gene sets related using DisGeNET which further improves the validity of the mouse-derived results.

However, we acknowledge some potential limitations in our study. First, our preclinical data are obtained using a murine model of AD that could not replicate entirely the neuropathological events observed in humans. Most behavioural differences between APP/PSEN1-Tg transgenic and non-transgenic mice were found at 6 months old around the onset of plaque pathology. One of our concerns is that we have carried out the behavioural experiments only in male mice, so further studies are required to investigate whether the emotional and cognitive alterations show similar time-course in females. We employed male mice in this specific APP/PSEN1-Tg mouse model because female mice develop higher β-amyloid plaque deposition than males [106], and it could interfere and shorten the time window to evaluate the behavioural experiments.

By contrast, the transcriptomic analysis revealed that most of the deregulated genes were observed at 3 months old when behavioural features were mildly altered. This will be investigated in ongoing follow-up studies. Second, despite we are using a widely used mouse model of AD (B6C3-Tg (APPswe, PSEN1dD9)85Dbo/MmJax), there are more murine models that could develop either the same or other phenotypic features of the AD. In fact, the present mouse model for AD recapitulates the early-onset form of the disease, which is generally related to genetic factors [11, 14], instead of late-onset AD cases that represent the most part of AD manifestations, that involved both genetic and environmental factors [10]. Finally, we would like to mention some of the methodological limitations of the GSEA analysis. GSEA is a method that accounts for the gene–gene correlation structure but does not really correct for overlapping gene sets. This is why we have used the Jaccard index to remove the most overlapping gene sets. Also, neither the topology of the gene sets nor the weights of the belonging genes are not considered. And finally, the assessment of the statistical significance and correction of the method has also been questioned [107].

## Conclusions

We suggest that a progressive cognitive decline could be a putative indicator of the early onset of AD, including not only depression but also anxiety, spatial and working memory impairments and odour dysfunction, among others. The APP/PSEN1-Tg model of AD recapitulates the early-depressive, early-anxiety and other early-cognitive impairment manifestations before developing AD, as a prodromal stage of the neurodegenerative disease. Moreover, this cognitive decline seems to be intimately linked to transcriptomic and progressive changes in the brain. This is for the first time in our knowledge that four different brain areas are analysed obtaining transcriptomic dysregulation, which captures dynamic changes in the gene regulation before the onset of AD in a mouse model. In fact, this model provides *App*, *Prnp*, *Ciart* and *Dbp* as possible shared biomarkers of AD and MD in all four brain analysed regions, despite the implications of *App* and *Prnp* in MD remaining unclear [70, 108, 109]. However, there are some dysregulated genes in a region-specific manner that could also be referred as biomarker, including *Cdh23*, *Ptx4* and *Mc4r* genes in the hippocampus and the clock genes *Nr1d1* and *Bhlhe41*, *Maml3*, *Zap70*, *Itgax* and *App* in PFC of transgenic mice.

Furthermore, studies combining post-mortem human tissues of PFC, striatum, hippocampus and amygdala from AD patients with and without an early history of depression, and transcriptomic analysis could help to provide additional evidence on the combined biological signature of both diseases. In order to evaluate if the results found in this animal model recapitulate the relationship between AD and MD in the clinical setting, it would be interesting to perform an analysis in human subjects, using information contained in resources such as BIOCARD.

## Supplementary Information

**Additional file 1: Supplementary material and methods. Figure S1.** Hyperlocomotion and impairments in spatial working memory in APP/PSEN1-Tg mice. **Figure S2.** The anxiety-like behaviour is increased in APP/PSEN1-Tg mice. **Figure S3.** Comparison of disease-specific gene sets differentially expressed in APP/PSEN1-Tg mice in PFC, striatum, hippocampus and amygdala.

**Additional file 2.**

**Additional file 3.**

**Additional file 4.**

**Additional file 5.**

**Additional file 6.**

**Additional file 7.**

**Additional file 8.**

**Additional file 9.**

**Additional file 10.**

## Data Availability

The data that support the findings of the current study are available from the corresponding author on reasonable request.

## Abbreviations

AD: Alzheimer’s disease
APP: Beta-amyloid precursor protein
BSPD: Behavioural and psychological symptoms of dementia
DE: Differential expression
EPM: Elevated plus maze
FC: Fold change
FDR: False discovery rate
GSEA: Gene Set Enrichment Analysis
GWAS: Genome-wide association study
ICD-9: International Classification of Diseases, 9th Edition
MD: Major depression
NES: Normalized enrichment score
NOR: Novel object recognition
OF: Open field
PFC: Prefrontal cortex
PSEN1: Presenilin-1
TST: Tail suspension test

## References

[1] World Health Organization (2017). Depression and other common mental disorders.

[2] Alexopoulos GS, Meyers BS, Young RC, Kalayam B, Kakuma T, Gabrielle M, Sirey JA, Hull J (2000). Executive dysfunction and long-term outcomes of geriatric depression. Arch Gen Psychiatry.

[3] Potter GG, Kittinger JD, Wagner HR, Steffens DC, Krishnan KRR (2004). Prefrontal neuropsychological predictors of treatment remission in late-life depression. Neuropsychopharmacology.

[4] Howard DM, Adams MJ, Clarke TK, Hafferty JD, Gibson J, Shirali M (2019). Genome-wide meta-analysis of depression identifies 102 independent variants and highlights the importance of the prefrontal brain regions. Nat Neurosci.

[5] Wray NR, Ripke S, Mattheisen M, Trzaskowski M, Byrne EM, Abdellaoui A (2018). Genome-wide association analyses identify 44 risk variants and refine the genetic architecture of major depression. Nat Genet.

[6] Ownby RL, Crocco E, Acevedo A, John V, Loewenstein D (2006). Depression and risk for Alzheimer disease: systematic review, meta-analysis, and metaregression analysis. Arch Gen Psychiatry.

[7] Kaup AR, Byers AL, Falvey C, Simonsick EM, Satterfield S, Ayonayon HN, Smagula SF, Rubin SM, Yaffe K (2016). Trajectories of depressive symptoms in older adults and risk of dementia. JAMA Psychiatry.

[8] Holmquist S, Nordström A, Nordström P. The association of depression with subsequent dementia diagnosis: a Swedish nationwide cohort study from 1964 to 2016. Brayne C, editor. PLoS Med. 2020;17:e1003016. 10.1371/journal.pmed.1003016 PMC6952081 31917808

[9] World Health Organization (2017). WHO Global action plan on the public health response to dementia 2017–2025.

[10] Sims R, Hill M, Williams J (2020). The multiplex model of the genetics of Alzheimer’s disease. Nat Neurosci.

[11] Belloy ME, Napolioni V, Greicius MD (2019). A quarter century of APOE and Alzheimer’s disease: progress to date and the path forward. Neuron..

[12] Krasemann S, Madore C, Cialic R, Baufeld C, Calcagno N, El Fatimy R (2017). The TREM2-APOE pathway drives the transcriptional phenotype of dysfunctional microglia in neurodegenerative diseases. Immunity.

[13] Mahley RW, Rall SC (2000). Apolipoprotein E: far more than a lipid transport protein. Annu Rev Genomics Hum Genet.

[14] Wijsman EM, Daw EW, Yu X, Steinbart EJ, Nochlin D, Bird TB (2005). APOE and other loci affect age-at-onset in Alzheimer’s disease families with PS2 mutation. Am J Med Genet Neuropsychiatr Genet.

[15] Van Duijn CM, de Knijff P, Cruts M, Wehnert A, Havekes LM, Hofman A (1994). Apolipoprotein E4 allele in a population–based study of early–onset Alzheimer’s disease. Nat Genet.

[16] Coppus AMW, Evenhuis HM, Verberne GJ, Visser FE, Arias-Vasquez A, Sayed-Tabatabaei FA, Vergeer-Drop J, Eikelenboom P, van Gool WA, van Duijn CM (2008). The impact of apolipoprotein E on dementia in persons with Down’s syndrome. Neurobiol Aging.

[17] Wang Y, Cella M, Mallinson K, Ulrich JD, Young KL, Robinette ML (2015). TREM2 lipid sensing sustains the microglial response in an Alzheimer’s disease model. Cell.

[18] El Khoury J, Toft M, Hickman SE, Means TK, Terada K, Geula C (2007). Ccr2 deficiency impairs microglial accumulation and accelerates progression of Alzheimer-like disease. Nat Med.

[19] van der Linde RM, Dening T, Stephan BCM, Prina AM, Evans E, Brayne C (2016). Longitudinal course of behavioural and psychological symptoms of dementia: systematic review. Br J Psychiatry.

[20] Dorey JM, Beauchet O, Anterion CT, Rouch I, Krolak-Salmon P, Gaucher J, et al. Behavioral and psychological symptoms of dementia and bipolar spectrum disorders: review of the evidence of a relationship and treatment implications. CNS Spectr. 2008;13(9):796–803. MBL Communications. 10.1017/s1092852900013924. 10.1017/s1092852900013924 18849899

[21] Dowlati Y, Herrmann N, Swardfager W, Liu H, Sham L, Reim EK, Lanctôt KL (2010). A meta-analysis of cytokines in major depression. Biol Psychiatry.

[22] White CS, Lawrence CB, Brough D, Rivers-Auty J (2017). Inflammasomes as therapeutic targets for Alzheimer’s disease. Brain Pathol.

[23] Rodrigues R, Petersen RB, Perry G (2014). Parallels between major depressive disorder and Alzheimer’s disease: role of oxidative stress and genetic vulnerability. Cell Mol Neurobiol.

[24] Song W, Hooli B, Mullin K, Jin SC, Cella M, Ulland TK (2017). Alzheimer’s disease-associated TREM2 variants exhibit either decreased or increased ligand-dependent activation. Alzheimer’s Dement.

[25] Pereira AC, Gray JD, Kogan JF, Davidson RL, Rubin TG, Okamoto M, Morrison JH, McEwen BS (2017). Age and Alzheimer’s disease gene expression profiles reversed by the glutamate modulator riluzole. Mol Psychiatry.

[26] Klaassens BL, van Gerven JMA, Klaassen ES, van der Grond J, Rombouts SARB (2019). Cholinergic and serotonergic modulation of resting state functional brain connectivity in Alzheimer’s disease. Neuroimage..

[27] Sullivan PF, Daly MJ, O’Donovan M (2012). Genetic architectures of psychiatric disorders: the emerging picture and its implications. Nat Rev Genet.

[28] Porcelli S, Salfi R, Politis A, Atti AR, Albani D, Chierchia A, Polito L, Zisaki A, Piperi C, Liappas I, Alberti S, Balestri M, Marsano A, Stamouli E, Mailis A, Biella G, Forloni G, Bernabei V, Ferrari B, Lia L, Papadimitriou GN, de Ronchi D, Serretti A (2013). Association between Sirtuin 2 gene rs10410544 polymorphism and depression in Alzheimer’s disease in two independent European samples. J Neural Transm.

[29] Webster SJ, Bachstetter AD, Nelson PT, Schmitt FA, Van Eldik LJ (2014). Using mice to model Alzheimer’s dementia: an overview of the clinical disease and the preclinical behavioral changes in 10 mouse models. Front Genet.

[30] Du Y, Du Y, Zhang Y, Huang Z, Fu M, Li J (2019). MKP-1 reduces Aβ generation and alleviates cognitive impairments in Alzheimer’s disease models. Signal Transduct Target Ther.

[31] Jankowsky JL, Slunt HH, Gonzales V, Jenkins NA, Copeland NG, Borchelt DR (2004). APP processing and amyloid deposition in mice haplo-insufficient for presenilin 1. Neurobiol Aging.

[32] Gracia-Rubio I, Moscoso-Castro M, Pozo OJ, Marcos J, Nadal R, Valverde O (2016). Maternal separation induces neuroinflammation and long-lasting emotional alterations in mice. Prog Neuropsychopharmacol Biol Psychiatry.

[33] Montagud-Romero S, Daza-Losada M, Vidal-Infer A, Maldonado C, Aguilar MA, Miñarro J (2014). The novelty-seeking phenotype modulates the long-lasting effects of intermittent ethanol administration during adolescence. Trezza V, editor. PLoS One.

[34] Filali M, Lalonde R, Rivest S (2009). Cognitive and non-cognitive behaviors in an APPswe/PS1 bigenic model of Alzheimer’s disease. Genes Brain Behav.

[35] Agustín-Pavón C, Martínez-Ricós J, Martínez-García F, Lanuza E (2009). Role of nitric oxide in pheromone-mediated intraspecific communication in mice. Physiol Behav.

[36] Paxinos G, Franklin KBJ. The mouse brain in stereotaxic coordinates. Academic. 2004;

[37] Lacinova Z, Dolinkova M, Haluzikova D, Krajickova J, Haluzik M (2008). Comparison of manual and automatic (MagNA Pure) isolation methods of total RNA from adipose tissue. Mol Biotechnol.

[38] Dobin A, Davis CA, Schlesinger F, Drenkow J, Zaleski C, Jha S, Batut P, Chaisson M, Gingeras TR (2013). STAR: ultrafast universal RNA-seq aligner. Bioinformatics..

[39] Robinson MD, Oshlack A (2010). A scaling normalization method for differential expression analysis of RNA-seq data. Genome Biol.

[40] Robinson MD, McCarthy DJ, Smyth GK (2010). edgeR: a Bioconductor package for differential expression analysis of digital gene expression data. Bioinformatics..

[41] Smyth GK (2005). limma: linear models for microarray data. Bioinforma Comput Biol Solut using R Bioconductor.

[42] Rojo AI, Pajares M, Rada P, Nuñez A, Nevado-Holgado AJ, Killik R, van Leuven F, Ribe E, Lovestone S, Yamamoto M, Cuadrado A (2017). NRF2 deficiency replicates transcriptomic changes in Alzheimer’s patients and worsens APP and TAU pathology. Redox Biol.

[43] Vandesompele J, De Preter K, Pattyn F, Poppe B, Van Roy N, De Paepe A, et al. Accurate normalization of real-time quantitative RT-PCR data by geometric averaging of multiple internal control genes. Genome Biol Genome Biol. 2002;3(7)research0034.1–research0034.11. 10.1186/gb-2002-3-7-research0034. 10.1186/gb-2002-3-7-research0034 PMC126239 12184808

[44] Subramanian A, Tamayo P, Mootha VK, Mukherjee S, Ebert BL, Gillette MA, Paulovich A, Pomeroy SL, Golub TR, Lander ES, Mesirov JP (2005). Gene set enrichment analysis: a knowledge-based approach for interpreting genome-wide expression profiles. Proc Natl Acad Sci U S A.

[45] Lai L, Hennessey J, Bares V, Son EW, Ban Y, Wang W, et al. GSKB: a gene set database for pathway analysis in mouse. bioRxiv. 2016. 10.1101/082511.

[46] Piñero J, Ramírez-Anguita JM, Saüch-Pitarch J, Ronzano F, Centeno E, Sanz F, Furlong LI (2020). The DisGeNET knowledge platform for disease genomics: 2019 update. Nucleic Acids Res.

[47] Wirz KTS, Bossers K, Stargardt A, Kamphuis W, Swaab DF, Hol EM, Verhaagen J (2013). Cortical beta amyloid protein triggers an immune response, but no synaptic changes in the APPswe/PS1dE9 Alzheimer’s disease mouse model. Neurobiol Aging.

[48] Chaney A, Bauer M, Bochicchio D, Smigova A, Kassiou M, Davies KE (2018). Longitudinal investigation of neuroinflammation and metabolite profiles in the APP swe ×PS1 Δe9 transgenic mouse model of Alzheimer’s disease. J Neurochem.

[49] Langa KM, Levine DA (2014). The diagnosis and management of mild cognitive impairment: a clinical review. JAMA.

[50] Corrêa MS, Vedovelli K, Giacobbo BL, de Souza CEB, Ferrari P, de Lima Argimon II, Walz JC, Kapczinski F, Bromberg E (2015). Psychophysiological correlates of cognitive deficits in family caregivers of patients with Alzheimer disease. Neuroscience..

[51] Attems J, Walker L, Jellinger KA (2014). Olfactory bulb involvement in neurodegenerative diseases. Acta Neuropathol.

[52] Burrage E, Marshall K, Santanam N, Chantler P (2018). Cerebrovascular dysfunction with stress and depression. Brain Circ.

[53] Taylor WD, Aizenstein HJ, Alexopoulos GS. The vascular depression hypothesis: mechanisms linking vascular disease with depression. Mol Psychiatry. 2013;18:963–74. NIH Public Access. 10.1038/mp.2013.20. 10.1038/mp.2013.20 PMC3674224 23439482

[54] Johansson M, Stomrud E, Lindberg O, Westman E, Johansson PM, van Westen D (2020). Apathy and anxiety are early markers of Alzheimer’s disease. Neurobiol Aging.

[55] Baillon S, Gasper A, Wilson-Morkeh F, Pritchard M, Jesu A, Velayudhan L (2019). Prevalence and severity of neuropsychiatric symptoms in early- versus late-onset Alzheimer’s disease. Am J Alzheimers Dis Other Demen.

[56] Ferreira M d C, Abreu MJ, Machado C, Santos B, Machado Á, Costa AS (2018). Neuropsychiatric profile in early versus late onset Alzheimer’s disease. Am J Alzheimers Dis Other Demen.

[57] Kirova AM, Bays RB, Lagalwar S. Working memory and executive function decline across normal aging, mild cognitive impairment, and Alzheimer’s disease. Biomed Res Int. 2015;2015:748212. Hindawi Limited. 10.1155/2015/748212. 10.1155/2015/748212 PMC4624908 26550575

[58] Goldstein FC, Loring DW, Thomas T, Saleh S, Hajjar I (2019). Recognition memory performance as a cognitive marker of prodromal Alzheimer’s disease. J Alzheimers Dis.

[59] Coughlan G, Laczó J, Hort J, Minihane AM, Hornberger M (2018). Spatial navigation deficits — overlooked cognitive marker for preclinical Alzheimer disease?. Nat Rev Neurol.

[60] Doi T, Shimada H, Makizako H, Tsutsumimoto K, Uemura K, Suzuki T (2015). Apolipoprotein E genotype and physical function among older people with mild cognitive impairment. Geriatr Gerontol Int.

[61] Wilson RS, Arnold SE, Schneider JA, Boyle PA, Buchman AS, Bennett DA. Olfactory impairment in presymptomatic Alzheimer’s disease. Ann N Y Acad Sci. 2009;1170:730–5. Blackwell Publishing Inc. 10.1111/j.1749-6632.2009.04013.x. 10.1111/j.1749-6632.2009.04013.x PMC2857767 19686220

[62] Ally BA. Using pictures and words to understand recognition memory deterioration in amnestic mild cognitive impairment and Alzheimer’s disease: a review. Curr Neurol Neurosci Rep. 2012;12(6):687–94. 10.1007/s11910-012-0310-7. 10.1007/s11910-012-0310-7 PMC3483366 22927024

[63] Satyanarayanan SK, Chien Y-C, Chang JP-C, Huang S-Y, Guu T-W, Su H (2020). Melatonergic agonist regulates circadian clock genes and peripheral inflammatory and neuroplasticity markers in patients with depression and anxiety. Brain Behav Immun.

[64] Li JZ, Bunney BG, Meng F, Hagenauer MH, Walsh DM, Vawter MP, Evans SJ, Choudary PV, Cartagena P, Barchas JD, Schatzberg AF, Jones EG, Myers RM, Watson SJ, Akil H, Bunney WE (2013). Circadian patterns of gene expression in the human brain and disruption in major depressive disorder. Proc Natl Acad Sci U S A.

[65] Cao X, Li LP, Wang Q, Wu Q, Hu HH, Zhang M, Fang YY, Zhang J, Li SJ, Xiong WC, Yan HC, Gao YB, Liu JH, Li XW, Sun LR, Zeng YN, Zhu XH, Gao TM (2013). Astrocyte-derived ATP modulates depressive-like behaviors. Nat Med.

[66] Galeotti N, Ghelardini C (2011). Antidepressant phenotype by inhibiting the phospholipase Cβ1 - protein kinase Cγ pathway in the forced swim test. Neuropharmacology.

[67] Ganea K, Menke A, Schmidt MV, Lucae S, Rammes G, Liebl C, Harbich D, Sterlemann V, Storch C, Uhr M, Holsboer F, Binder EB, Sillaber I, Müller MB (2012). Convergent animal and human evidence suggests the activin/inhibin pathway to be involved in antidepressant response. Transl Psychiatry.

[68] Chaki S, Okuyama S (2005). Involvement of melanocortin-4 receptor in anxiety and depression. Peptides.

[69] Dean B, Tsatsanis A, Lam LQ, Scarr E, Duce JA (2019). Changes in cortical protein markers of iron transport with gender, major depressive disorder and suicide. World J Biol Psychiatry.

[70] Watanabe S, Iga J, Ishii K, Numata S, Shimodera S, Fujita H (2015). Biological tests for major depressive disorder that involve leukocyte gene expression assays. J Psychiatr Res.

[71] Lobão-Soares B, Walz R, Carlotti CG, Sakamoto AC, Calvo F, Terzian ALB (2007). Cellular prion protein regulates the motor behaviour performance and anxiety-induced responses in genetically modified mice. Behav Brain Res.

[72] Beckman D, Santos LE, Americo TA, Ledo JH, De Mello FG, Linden R (2015). Prion protein modulates monoaminergic systems and depressive-like behavior in mice. J Biol Chem.

[73] Petersen RC, Smith GE, Waring SC, Ivnik RJ, Tangalos EG, Kokmen E (1999). Mild cognitive impairment: clinical characterization and outcome. Arch Neurol.

[74] De Jager PL, Ma Y, McCabe C, Xu J, Vardarajan BN, Felsky D (2018). Data descriptor: a multi-omic atlas of the human frontal cortex for aging and Alzheimer’s disease research. Sci Data.

[75] White CC, Yang HS, Yu L, Chibnik LB, Dawe RJ, Yang J, et al. Identification of genes associated with dissociation of cognitive performance and neuropathological burden: multistep analysis of genetic, epigenetic, and transcriptional data. PLoS Med. 2017;14(4):e1002287. Public Libr Sci. 10.1371/journal.pmed.1002287. 10.1371/journal.pmed.1002287 PMC5404753 28441426

[76] Soldan A, Pettigrew C, Cai Q, Wang J, Wang MC, Moghekar A (2017). Cognitive reserve and long-term change in cognition in aging and preclinical Alzheimer’s disease. Neurobiol Aging.

[77] Albert M, Soldan A, Gottesman R, McKhann G, Sacktor N, Farrington L (2014). Cognitive changes preceding clinical symptom onset of mild cognitive impairment and relationship to ApoE genotype. Curr Alzheimer Res.

[78] Chan CK, Soldan A, Pettigrew C, Wang J, Albert M, Rosenberg PB. Depressive symptoms and CSF Alzheimer’s disease biomarkers in relation to clinical symptom onset of mild cognitive impairment. Alzheimers Dement Diagn Assess Dis Monit. 2020;12(1):e12106. Wiley. 10.1002/dad2.12106. 10.1002/dad2.12106 PMC7513625 33005725

[79] Mills F, Bartlett TE, Dissing-Olesen L, Wisniewska MB, Kuznicki J, Macvicar BA, Wang YT, Bamji SX (2014). Cognitive flexibility and long-term depression (LTD) are impaired following β-catenin stabilization in vivo. Proc Natl Acad Sci U S A.

[80] Bjartmar L, Alkhori L, Ruud J, Mohammed AH, Marcusson J, Hallbeck M (2010). Long-term treatment with antidepressants, but not environmental stimulation, induces expression of NP2 mRNA in hippocampus and medial habenula. Brain Res.

[81] Shen Y, Tian M, Zheng Y, Gong F, Fu AKY, Ip NY (2016). Stimulation of the hippocampal POMC/MC4R circuit alleviates synaptic plasticity impairment in an Alzheimer’s disease model. Cell Rep.

[82] Serova LI, Laukova M, Alaluf LG, Sabban EL (2014). Blockage of melanocortin-4 receptors by intranasal HS014 attenuates single prolonged stress-triggered changes in several brain regions. J Neurochem.

[83] Goldman-Rakic P (1995). Cellular basis of working memory. Neuron..

[84] Zhong P, Gu Z, Wang X, Jiang H, Feng J, Yan Z (2003). Impaired modulation of GABAergic transmission by muscarinic receptors in a mouse transgenic model of Alzheimer’s disease. J Biol Chem.

[85] Chapman PF, White GL, Jones MW, Cooper-Blacketer D, Marshall VJ, Irizarry M, Younkin L, Good MA, Bliss TVP, Hyman BT, Younkin SG, Hsiao KK (1999). Impaired synaptic plasticity and learning in aged amyloid precursor protein transgenic mice. Nat Neurosci.

[86] Hsiao K, Chapman P, Nilsen S, Eckman C, Harigaya Y, Younkin S (1996). Correlative memory deficits, Aβ elevation, and amyloid plaques in transgenic mice. Science (80- ).

[87] Finnie GS, Gunnarsson R, Manavis J, Blumbergs PC, Mander KA, Edwards S, van den Heuvel C, Finnie JW (2017). Characterization of an ‘amyloid only’ transgenic (B6C3-Tg (APPswe,PSEN1dE9)85Dbo/Mmjax) mouse model of Alzheimer’s disease. J Comp Pathol.

[88] Chen B, Zhong X, Mai N, Peng Q, Wu Z, Ouyang C, Zhang W, Liang W, Wu Y, Liu S, Chen L, Ning Y (2018). Cognitive impairment and structural abnormalities in late life depression with olfactory identification impairment: an Alzheimer’s disease-like pattern. Int J Neuropsychopharmacol.

[89] Barger SW, Harmon AD (1997). Microglial activation by Alzheimer amyloid precursor protein and modulation by apolipoprotein E. Nature.

[90] Ho Kim J, Franck J, Kang T, Heinsen H, Ravid R, Ferrer I (2015). Proteome-wide characterization of signalling interactions in the hippocampal CA4/DG subfield of patients with Alzheimer’s disease. Sci Rep.

[91] Nanus DE, Wijesinghe SN, Pearson MJ, Hadjicharalambous MR, Rosser A, Davis ET (2020). Regulation of the inflammatory synovial fibroblast phenotype by metastasis-associated lung adenocarcinoma transcript 1 long noncoding RNA in obese patients with osteoarthritis. Arthritis Rheumatol.

[92] He W, Yuan T, Maedler K (2019). Macrophage-associated pro-inflammatory state in human islets from obese individuals. Nutr Diabetes.

[93] Shay T, Jojic V, Zuk O, Rothamel K, Puyraimond-Zemmour D, Feng T, Wakamatsu E, Benoist C, Koller D, Regev A, the ImmGen Consortium (2013). Conservation and divergence in the transcriptional programs of the human and mouse immune systems. Proc Natl Acad Sci U S A.

[94] Mestas J, Hughes CCW (2004). Of mice and not men: differences between mouse and human immunology. J Immunol.

[95] Holper L, Ben-Shachar D, Mann J. Multivariate meta-analyses of mitochondrial complex I and IV in major depressive disorder, bipolar disorder, schizophrenia, Alzheimer disease, and Parkinson disease. Neuropsychopharmacology. 2019;44(5):837–49. Nature Publishing Group. 10.1038/s41386-018-0090-0. 10.1038/s41386-018-0090-0 PMC6461987 29855563

[96] Czarny P, Wigner P, Galecki P, Sliwinski T. The interplay between inflammation, oxidative stress, DNA damage, DNA repair and mitochondrial dysfunction in depression. Prog Neuropsychopharmacol Biol Psychiatry. 2018;80(Pt C):309–21. Elsevier Inc. 10.1016/j.pnpbp.2017.06.036. 10.1016/j.pnpbp.2017.06.036 28669580

[97] Sharma A, Sethi G, Tambuwala MM, Aljabali AAA, Chellappan DK, Dua K (2020). Circadian rhythm disruption and Alzheimer’s disease: the dynamics of a vicious cycle. Curr Neuropharmacol.

[98] Musiek ES, Holtzman DM. Mechanisms linking circadian clocks, sleep, and neurodegeneration. Science. 2016;354(6315):1004–8. American Association for the Advancement of Science. 10.1126/science.aah4968. 10.1126/science.aah4968 PMC5219881 27885006

[99] Orozco-Solis R, Montellier E, Aguilar-Arnal L, Sato S, Vawter MP, Bunney BG, Bunney WE, Sassone-Corsi P (2017). A circadian genomic signature common to ketamine and sleep deprivation in the anterior cingulate cortex. Biol Psychiatry.

[100] Difrancesco S, Lamers F, Riese H, Merikangas KR, Beekman ATF, van Hemert AM (2019). Sleep, circadian rhythm, and physical activity patterns in depressive and anxiety disorders: a 2-week ambulatory assessment study. Depress Anxiety.

[101] Irwin MR (2019). Sleep and inflammation: partners in sickness and in health. Nat Rev Immunol.

[102] Takashima A, Murayama M, Murayama O, Kohno T, Honda T, Yasutake K (1998). Presenilin 1 associates with glycogen synthase kinase-3β and its substrate tau. Proc Natl Acad Sci U S A.

[103] Lovestone S, Reynolds CH, Latimer D, Davis DR, Anderton BH, Gallo JM, Hanger D, Mulot S, Marquardt B, Stabel S, Woodgett JR, Miller CCJ (1994). Alzheimer’s disease-like phosphorylation of the microtubule-associated protein tau by glycogen synthase kinase-3 in transfected mammalian cells. Curr Biol.

[104] Hooper C, Killick R, Lovestone S (2008). The GSK3 hypothesis of Alzheimer’s disease. J Neurochem.

[105] Iitaka C, Miyazaki K, Akaike T, Ishida N (2005). A role for glycogen synthase kinase-3β in the mammalian circadian clock. J Biol Chem.

[106] Wang J, Tanila H, Puoliväli J, Kadish I, Van Groen T (2003). Gender differences in the amount and deposition of amyloidβ in APPswe and PS1 double transgenic mice. Neurobiol Dis.

[107] Simillion C, Liechti R, Lischer HEL, Ioannidis V, Bruggmann R. Avoiding the pitfalls of gene set enrichment analysis with SetRank. BMC Bioinformatics. 2017;18(1):151. BioMed Central Ltd. 10.1186/s12859-017-1571-6. 10.1186/s12859-017-1571-6 PMC5336655 28259142

[108] Inoue M, Baba H, Yamamoto K, Shimada H, Yamakawa Y, Suzuki T (2016). Serum levels of albumin–β-amyloid complex in patients with depression. Am J Geriatr Psychiatry.

[109] Harrington KD, Lim YY, Gould E, Maruff P (2015). Amyloid-beta and depression in healthy older adults: a systematic review. Aust N Z J Psychiatry.

