# Supplementary material for: Comorbidity between Alzheimer’s disease and major depression: a behavioural and transcriptomic characterization study in mice
Source: Alzheimers Res Ther. 2021 Apr 2;13:73. doi: 10.1186/s13195-021-00810-x (PMC8017643; doi:10.1186/s13195-021-00810-x)
Supplement: Supplementary file 1 — Additional file 1: Supplementary material and methods. Figure S1. Hyperlocomotion and impairments in spatial working memory in APP/PSEN1-Tg mice. Figure S2. The anxiety-like behaviour is increased in APP/PSEN1-Tg mice. Figure S3. Comparison of disease-specific gene sets differentially expressed in APP/PSEN1-Tg mice in PFC, striatum, hippocampus and amygdala. [file 13195_2021_810_MOESM1_ESM.docx]

**Comorbidity between Alzheimer’s disease and Major Depression: a behavioural and transcriptomic characterization study in mice**

Ana Martín-Sánchez^1^, Janet Piñero^2^, Lara Nonell^2,3^, Magdalena Arnal^2^, Elena M. Ribe^4^, Alejo Nevado-Holgado^4,5^, Simon Lovestone^4,6^, Ferran Sanz^2^, Laura I. Furlong^2^, Olga Valverde^1*^

^1.^ Neurobiology of Behaviour Research Group (GReNeC-NeuroBio), Department of Experimental and Health Sciences, Universitat Pompeu Fabra; Neuroscience Research Program, IMIM-Hospital del Mar Research Institute, Barcelona, Spain.

^2.^ Research Programme on Biomedical Informatics (GRIB), IMIM-Hospital del Mar Medical Research Institute, Universitat Pompeu Fabra, Barcelona, Spain.

^3.^ MARGenomics core facility, IMIM-Hospital del Mar Medical Research Institute, Barcelona, Spain.

^4.^ Department of Psychiatry, University of Oxford, Oxford OX3 7JX, UK

^5.^ Oxford Health NHS Foundation Trust, Oxford OX3 7JX, UK

^6^ currently at Janssen-Cilag, UK

^*^Correspondence should be addressed to:

Olga Valverde

Department of Experimental and Health Sciences (DCEXS)

Universitat Pompeu Fabra

Carrer Doctor Aiguader 88, Barcelona 08003 Spain

Tel: +34 93 316 08 67

E-mail: olga.valverde@upf.edu

**SUPPLEMENTARY MATERIAL AND METHODS**

*Locomotor activity.* We assessed the locomotor activity for 20min1. using the LE 8816 IR motor activity monitor (Panlab s.l.u., Barcelona, Spain). The test chambers (250 × 250 × 200 mm) had a black plastic floor and clear plastic walls, with two-dimensional (x- and y-axis) tracking sensors composed of 16 x 16 infrared beams for subject detection. During the test, the subjects were shielded from external noise and illuminated with indirect white light. Horizontal and vertical movements were measured from photocell beam interruptions using the SEDACOM software (Panlab s.l.u., Barcelona, Spain). Between trials, the apparatus was cleaned with 70% ethanol.

*Spontaneous alternation Y-maze.* Mice of both groups (APP/PSEN1-Tg and Non-Transgenic) at two different ages underwent for [spatial working memory](https://www.sciencedirect.com/topics/neuroscience/spatial-memory) test as previously reported^2^ We placed each mouse in the centre of a Y-shaped maze with the two equal arms, each 395 mm long and separated by 120° angles, and were allowed to freely explore for 8 min. Arm choices were manually recorded. Three consecutive choices of three different arms were counted as an alternation. The score was calculated by: [the total number of alternations] / [total number of choices - 2]. Additionally, the number of mistakes were also counted, considering a mistake as two consecutive entrances in the same arm. Between trials, the maze was cleaned with 70% ethanol.

*Open Field (OF).* The test was performed in a white Plexiglas box (500 × 500 × 300 mm) with white vertical walls under dim light intensity (30 lux) in the centre of the field. At the beginning of each test, mice were placed at the centre of the floor. Then, they were allowed to move freely around the maze and to explore the environment for 10 min. The video tracking software SMART (Panlab s.l.u., Spain) was used to measure the following parameters: the time that each mouse spent in the centre (125 × 125 mm), corners (125 × 125 mm each square in the corner) and surroundings (250 × 250 mm each peripheric area between two corners), the number visits of each and the total distance travelled. Between trials, the arena was cleaned with 70% ethanol.

**RESULTS**

*Hyperlocomotion behaviour in APP/PSEN1-Tg animals at 3 and 6 months old*

Two-way ANOVA for repeated measurements revealed a main *Genotype* effect (F_1,30_ = 6.27, p=0.018; Supplementary Figure 1A), *Time* effect (F_3,90_ = 14.458; p=0.000) but not *genotype × time* interaction (p>0.05) at 3 months of age. The post-hoc with Bonferroni’s correction for *Genotype* effect revealed that all transgenic mice performed higher horizontal movements than control mice. Then, we evaluated the horizontal movements using an ANOVA for repeated measurements to evaluate differences horizontal locomotion during four intervals of time, we observed a main *Genotype* effect (F_1,26_ = 8.80, p=0.006; Supplementary Figure 1D) and *time* (F_3,24_=3.25, p=0.039) at 6 months of age. This effect indicates that APP/PSEN1-Tg show higher horizontal movements than Non-Transgenic mice. However, mice did not differ statistically in the vertical movements at both ages (p>0.05; data not shown).

*Spatial working impairments in APP/PSEN1-Tg animals at 6 months old*

The t-Student’s test indicated that both groups of animals showed the same percentage of alternations during Y-maze test at 3 months old (Supplementary Figure 1B) without any error (Supplementary Figure 1C). At 6 months old, APP/PSEN1-Tg animals decreased the percentage of alternations in comparison with Non-Transgenic animals (t_26_=4.181, p=0.0003; Supplementary Figure 1E). The Fisher’s exact test revealed that a lower percentage of Non-Carrier (7%, Supplementary Figure 1F) males made less number of errors during the alternations than APP/PSEN1-Tg animals (71% ; p<0.001).


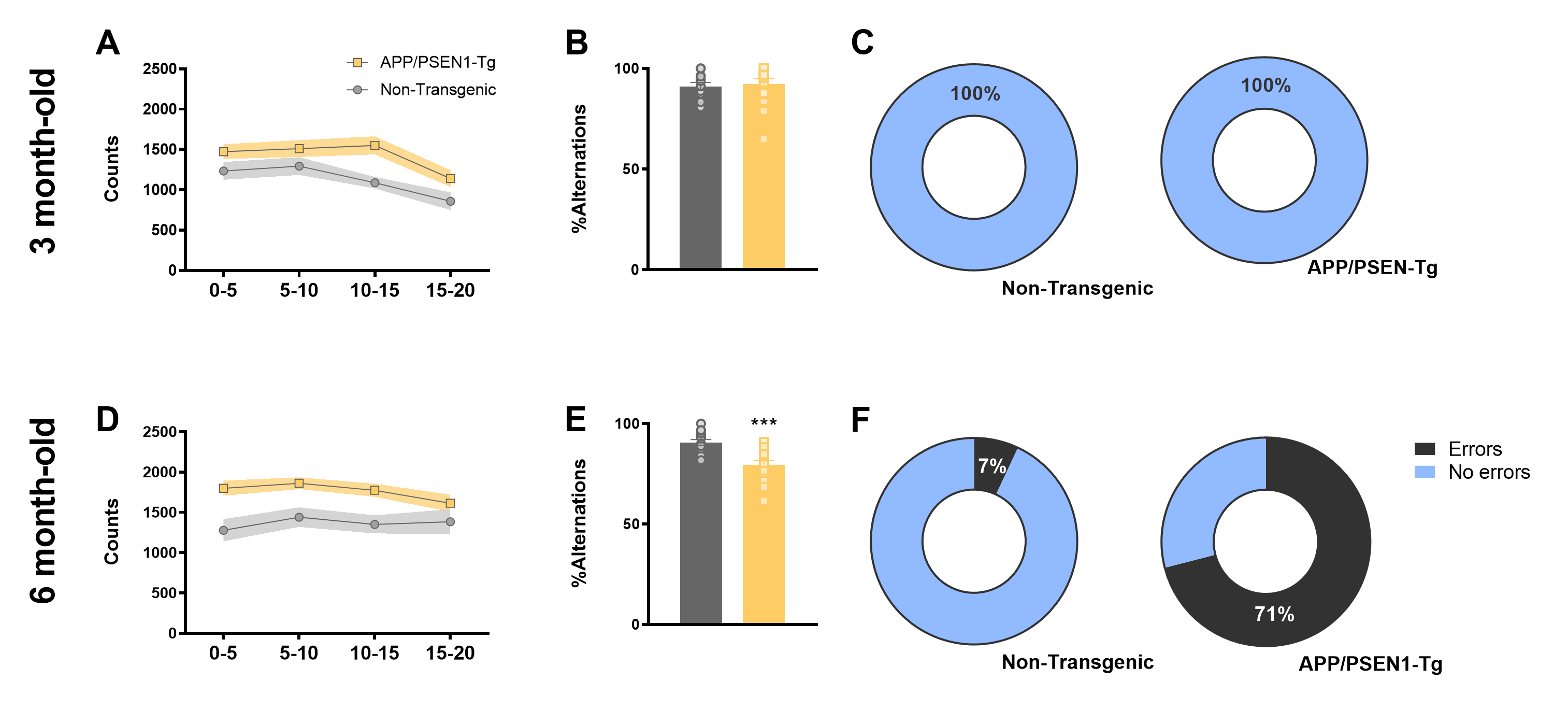


Figure S1. Hyperlocomotion and impairments in spatial working memory in APP/PSEN1-Tg mice. Data are presented as mean ± SEM (n=14-16). A and D represent the locomotion activity, whereas B,C, E and F show the Y-maze results. Squares represent the count of horizontal movements of Non-Transgenic animals whereas the circles represent total count of horizontal movements of APP/PSEN-Tg animals during five-minute intervals at 3 (A) and 6 month-old (D). The percentage of spontaneous alternations in Non-Transgenic (n=14-16) and APP/PSEN1-Tg (n=14-16) at 3- and 6-month-old (B, E). (***p<0.001, t-Student test Non-Transgenic vs APP/PSEN1-Tg). (C) 100% of Non-Transgenic and APP/PSEN1-Tg complete entirely the alternations without any mistake at 3 months old (blue area). (D) The 7% of Non-Transgenic animals (grey area) and the 71% of APP/PSEN1-Tg animals (grey area) made at least one mistake during Y-maze test (p<0.001, Fisher’s test).

*Open field*

When we evaluated the behaviour of mice in the open field, we observed that APP/PSEN1-Tg animals showed more entries in the surrounding areas (t_30_=2.355, p=0.0253; Supplementary Figure 2A at 3 months old; t_26_=2.913, p=0.0073; Supplementary Figure 2B at 6 months old) and more entries at the corners (t_30_=2.325, p=0.027; Supplementary Figure 2C at 3 months old; t_26_=2.790, p=0.0097 ; Supplementary Figure 2D at 6 months old). However, the data did not reveal differences in the number of entries at centre area (t_30_=0.657, p=0.516 and t_26_=1.839, p=0.077; Supplementary Figure 2E and F).

**
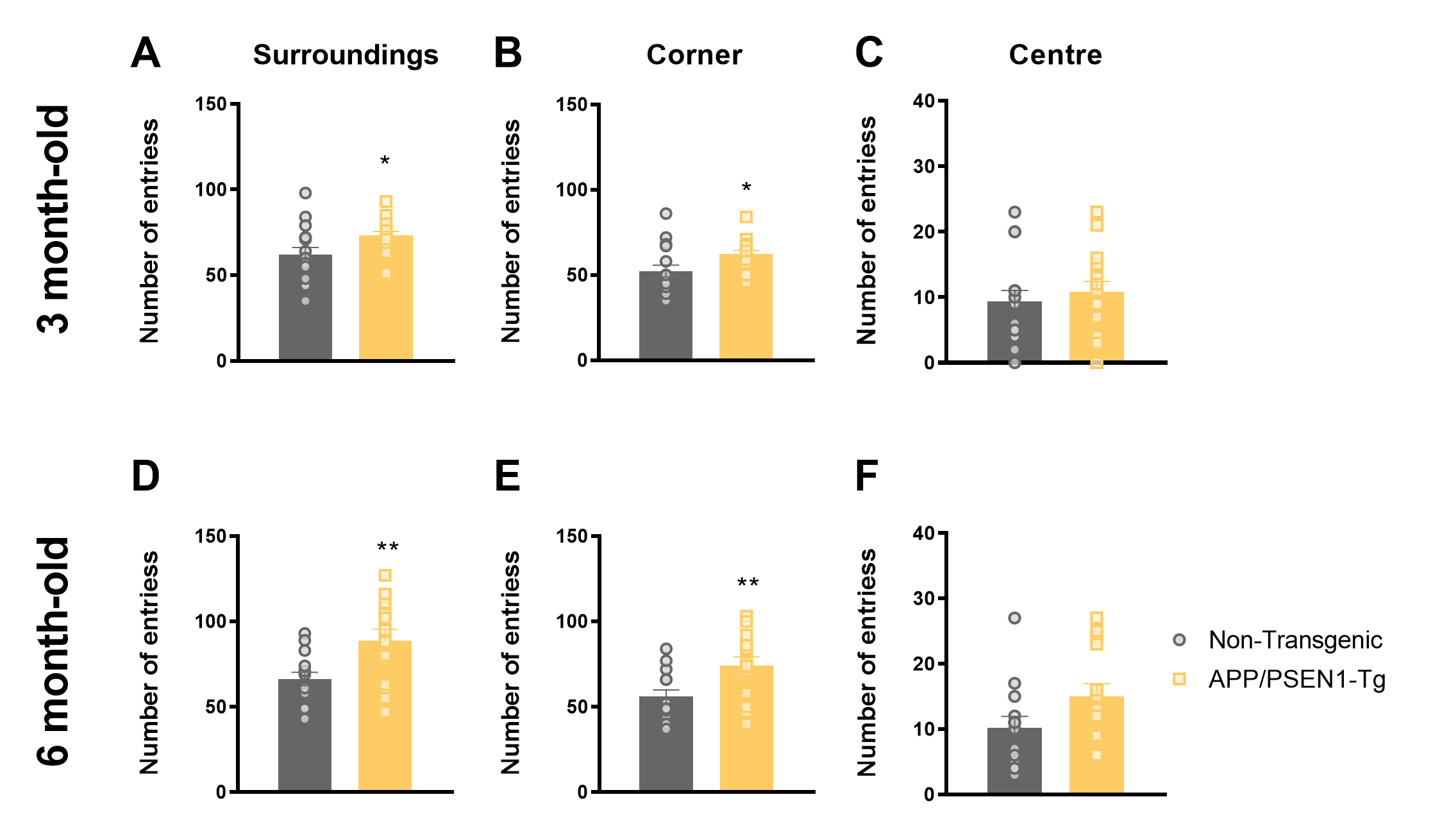
**

Figure S2. The anxiety-like behaviour is increased in APP/PSEN1-Tg mice. Grey (Non-Transgenic) and yellow (APP/PSEN1-Tg) bars represents the number of entries to the surroundings (A,D), corners (B,E) and the centre area (C,F) in the open field test at 3 (n=16/group) and 6 (n=14/group) month-old of age. Data are presented as mean ± SEM (n=14-16). *p<0.05, **p < 0.01 (t-Student test Non-Transgenic vs APP/PSEN1-Tg).


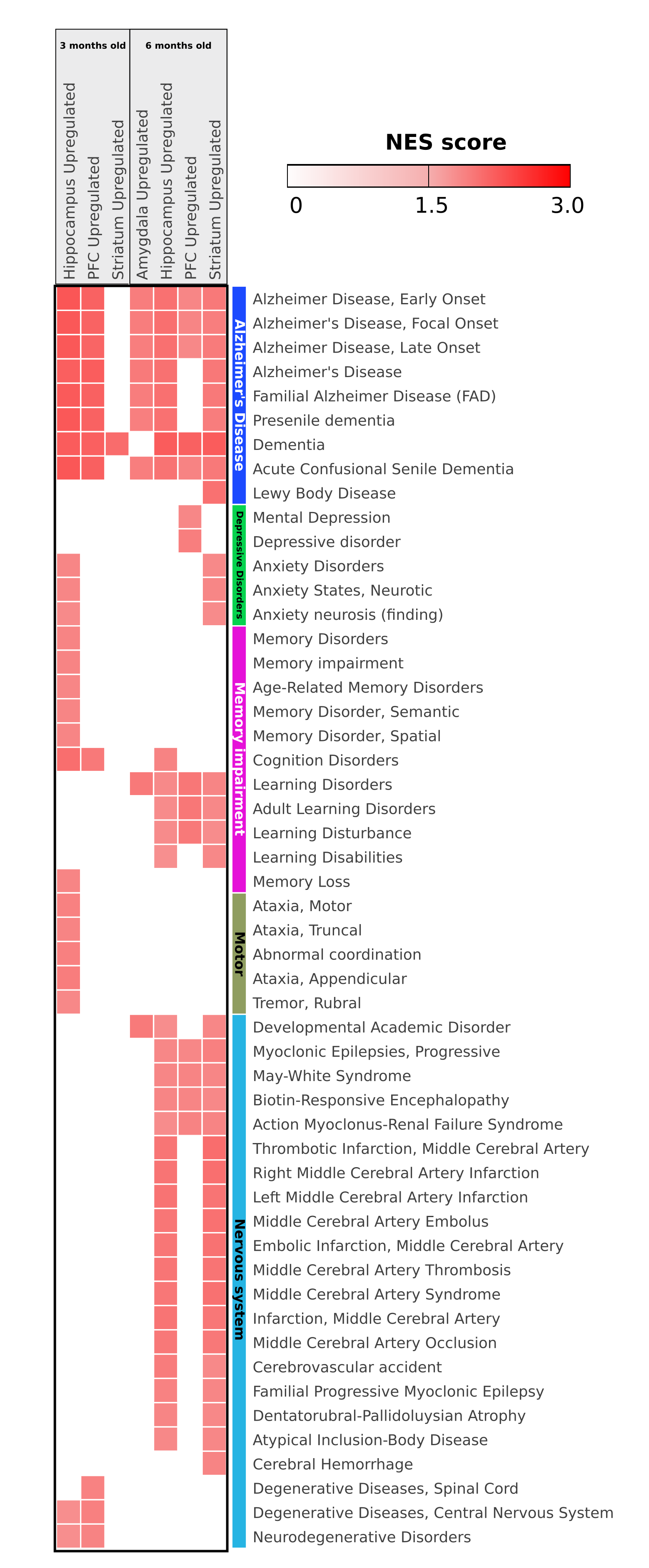


Figure S3. Comparison of disease-specific gene sets differentially expressed in APP/PSEN1-Tg mice in PFC, striatum, hippocampus and amygdala. The heatmap represents selected gene sets at 3 and 6 month-old upregulated in disease, and disease-related phenotypes, grouped in general categories: Alzheimer disease, Depressive disorders, Memory impairment, Motor impairment (Motor), and Nervous System Disorders (Nervous system). (FDR q value < 0.05; |NES| >1.4).

| Age | Brain area | Assay | RQ | pval | adj.pval |
| --- | --- | --- | --- | --- | --- |
| 3 months old | PFC | App-Mm01344172_m1 | 291.904 | 0,01478 | 9.61e-05 |
|  |  | Fosl2-Mm00484442_m1 | 133.191 | 0.04033 | 0.08738 |
|  |  | Maml3-Mm01294189_m1 | 161.919 | 0.001395 | 0.006046 |
|  |  | Inhba-Mm00434339_m1 | 130.063 | 0.02348 | 0.07632 |
|  |  | Prnp;Prn-Mm00448389_m1 | 691.253 | 1,21E-06 | 0,00001572 |
|  | Striatum | App-Mm01344172_m1 | 200.390 | 0.0008103 | 0.005267 |
|  |  | Fosl2-Mm00484442_m1 | 185.394 | 0.0115 | 0.04983 |
|  |  | Prnp;Prn-Mm00448389_m1 | 531.610 | 3,71E-08 | 4,83E-07 |
|  | Hippocampus | App-Mm01344172_m1 | 347826 | 0.00206 | 0.006696 |
|  |  | Olfr551-Mm00527595_s1 | 0.46600 | 0.005971 | 0.01552 |
|  |  | Maml3-Mm01294189_m1 | 132718 | 0.0006469 | 0.002803 |
|  |  | Itgax-Mm00498701_m1 | 183684 | 0.01229 | 0.02664 |
|  |  | Inhba-Mm00434339_m1 | 146468 | 0,09234 | 0.0006002 |
|  |  | Prnp;Prn-Mm00448389_m1 | 624330 | 5,47E-06 | 7,12E-05 |
|  |  | Edn1-Mm00438659_m1 | 135982 | 0.02278 | 0.0423 |
|  | Amygdala | Aldh1a3-Mm00474049_m1 | 359145 | 0.01497 | 0.03243 |
|  |  | Bglap3-Mm00649782_gH | 300451 | 0.01241 | 0.03226 |
|  |  | App-Mm01344172_m1 | 324314 | 0,001018 | 6,62E-03 |
|  |  | Dcn-Mm00514535_m1 | 445983 | 0.007065 | 0.02296 |
|  |  | Fosl2-Mm00484442_m1 | 180732 | 0.00213 | 0.00923 |
|  |  | Col4a2-Mm00802386_m1 | 127213 | 0.0267 | 0.04958 |
|  |  | Prnp;Prn-Mm00448389_m1 | 740368 | 5,09E-06 | 6,62E-05 |
| 6 monthd old | PFC | App-Mm01344172_m1 | 313.667 | 1.38e-06 | 8.97e-06 |
|  |  | Fosl2-Mm00484442_m1 | 187.721 | 0.001425 | 0.006175 |
|  |  | Col4a2-Mm00802386_m1 | 163.808 | 0.02058 | 0.03822 |
|  |  | Clec7a-Mm01183349_m1 | 465.260 | 0.002807 | 0.009124 |
|  |  | Maml3-Mm01294189_m1 | 150.494 | 0.0196 | 0.03822 |
|  |  | Inhba-Mm00434339_m1 | 133.208 | 0.01137 | 0.02957 |
|  |  | Prnp;Prn-Mm00448389_m1 | 867.955 | 3,99E-07 | 5,19E-06 |
|  | Striatum | App-Mm01344172_m1 | 279.421 | 2,09E-05 | 1,36E-04 |
|  |  | Clec7a-Mm01183349_m1 | 195.145 | 0.01211 | 0.03936 |
|  |  | Prnp;Prn-Mm00448389_m1 | 666.757 | 2,62E-06 | 3,40E-05 |
|  |  | Edn1-Mm00438659_m1 | 152.015 | 0.001162 | 0.005036 |
|  | Hippocampus | App-Mm01344172_m1 | 314.100 | 1,03E-05 | 6,69E-05 |
|  |  | Clec7a-Mm01183349_m1 | 372.467 | 0.0001063 | 0.0004608 |
|  |  | Prnp;Prn-Mm00448389_m1 | 750.823 | 1,92E-08 | 2,49E-07 |
|  | Amygdala | App-Mm01344172_m1 | 339.002 | 1,16E-05 | 7,55E-05 |
|  |  | Fosl2-Mm00484442_m1 | 148.411 | 0.008574 | 0.02229 |
|  |  | Clec7a-Mm01183349_m1 | 535.927 | 0.0001153 | 0.0004996 |
|  |  | Itgax-Mm00498701_m1 | 291.038 | 0.003691 | 0.012 |
|  |  | Prnp;Prn-Mm00448389_m1 | 727.674 | 1,04E-05 | 7,55E-05 |

***REFERENCES***

1. Ros-Simó, C., Ruiz-Medina, J. & Valverde, O. Behavioural and neuroinflammatory effects of the combination of binge ethanol and MDMA in mice. *Psychopharmacology (Berl).* **221**, 511–525 (2012).

2. Cantacorps, L. *et al.* Maternal alcohol binge drinking induces persistent neuroinflammation associated with myelin damage and behavioural dysfunctions in offspring mice. *Neuropharmacology* **123**, 368–384 (2017).
